# Developmental Changes in Composition and Morphology of Cuticular Waxes on Leaves and Spikes of Glossy and Glaucous Wheat (*Triticum aestivum* L.)

**DOI:** 10.1371/journal.pone.0141239

**Published:** 2015-10-27

**Authors:** Yong Wang, Jiahuan Wang, Guaiqiang Chai, Chunlian Li, Yingang Hu, Xinhong Chen, Zhonghua Wang

**Affiliations:** State Key Laboratory of Crop Stress Biology for Arid Areas, College of Agronomy, Northwest A&F University, Yangling, Shaanxi, China; Murdoch University, AUSTRALIA

## Abstract

The glossy varieties (A14 and Jing 2001) and glaucous varieties (Fanmai 5 and Shanken 99) of wheat (*Triticum aestivum* L.) were selected for evaluation of developmental changes in the composition and morphology of cuticular waxes on leaves and spikes. The results provide us with two different wax development patterns between leaf and spike. The general accumulation trend of the total wax load on leaf and spike surfaces is first to increase and then decrease during the development growth period, but these changes were caused by different compound classes between leaf and spike. Developmental changes of leaf waxes were mainly the result of variations in composition of alcohols and alkanes. In addition, diketones were the third important contributor to the leaf wax changes in the glaucous varieties. Alkanes and diketones were the two major compound classes that caused the developmental changes of spike waxes. For leaf waxes, β- and OH-β-diketones were first detected in flag leaves from 200-day-old plants, and the amounts of β- and OH-β-diketones were significantly higher in glaucous varieties compared with glossy varieties. In spike waxes, β-diketone existed in all varieties, but OH-β-diketone was detectable only in the glaucous varieties. Unexpectedly, the glaucous variety Fanmai 5 yielded large amounts of OH-β-diketone. There was a significant shift in the chain length distribution of alkanes between early stage leaf and flag leaf. Unlike C_28_ alcohol being the dominant chain length in leaf waxes, the dominant alcohol chain length of spikes was C_24_ or C_26_ depending on varieties. Epicuticular wax crystals on wheat leaf and glume were comprised of platelets and tubules, and the crystal morphology changed constantly throughout plant growth, especially the abaxial leaf crystals. Moreover, our results suggested that platelets and tubules on glume surfaces could be formed rapidly within a few days.

## Introduction

The leaf surfaces of land plants are covered by the cuticle, which forms the contact zone between the plant and the environment [1]. The cuticle has many functions. One principal function is to limit non-stomatal water loss, which contributes to drought tolerance [2–5]. In addition, the cuticle serves to separate adjacent organs during their development, acts as a defensive barrier against pests and pathogens [6, 7], provides protection from potentially damaging UV radiation [8], and participates in a variety of plant-insect interactions [9]. An unexpected function of wax has been reported by Preuss et al, who found that waxes in the tryphine-containing layer of pollen grains are essential for proper pollen-stigma interaction [10].

Structurally, the cuticle consists mainly of two hydrophobic components, the biopolymer cutin which is the main structural component of the cuticular matrix, and associated soluble lipids called cuticular waxes [11, 12]. Cutin is a three-dimensional polymer of mostly C_16_ and C_18_ hydroxy fatty acids cross-linked by ester bonds [13, 14], whereas cuticular wax is comprised of very-long-chain fatty acids (VLCFAs), primary and secondary fatty alcohols, ketones and aldehydes, alkanes, and esters, as well as other nonaliphatic components, such as triterpenoids and flavonoids [4, 15], with carbon chain lengths ranging from C_20_ to C_40_ in homologous series. Alkyl esters from C_38_ to C_70_ may also be present [16]. Wheat cuticular waxes comprise alcohols, alkanes, aldehydes, fatty acids, esters, β- and OH-β-diketones [17–20]. The chemical composition and amount of cuticular waxes varies greatly from species to species [21–23]. For example, the cuticular waxes on the leaves of Arabidopsis are mainly composed of alkanes, alcohols, and fatty acids [24]. In contrast, the waxes of maize seedling leaves are primarily alcohols, aldehydes and esters [25]. Even within the same plant, wax composition and amount varies between the different organs and tissues. In wheat, alcohols are the major components of wax from leaf blades and β-diketones are the major components of wax from leaf sheaths, especially the flag leaf sheath [26]. In Arabidopsis, the proportion of ketones in the leaf wax is 30-fold lower than the proportion of ketones in the stem wax [22]. Wax compositions also differed at the various developmental stages of the same organ. During the first 50 days after germination, the major component of wheat leaves is octacosanol. At 66 days, when sheath development is complete, β-diketone content is greatest [26]. Young maize leaves predominantly contain primary alcohols (63%), and older leaves predominantly contain wax esters (42%) [27, 28].

Cuticular wax components are specifically found as an epicuticular layer that covers leaves, fruits and young stems, giving the plant surface a glaucous or gray appearance [22, 29, 30]. In many plants, cuticular waxes are often present in the form of a smooth film or microcrystals. Since the introduction of scanning electron microscopy (SEM) in the 1960s, the microcrystal morphology of plant cuticular wax has been studied intensively [30]. Barthlott et al distinguished 23 wax types from at least 13, 000 species. Among these types, platelets and tubules are the most prominent [31]. Subsequently, Koch and Ensikat classified several common three-dimensional wax morphologies, including massive crusts, granules, plates, platelets, filaments, rods, and tubules with a hollow center [30]. In wheat, several studies over the past nine years have examined the wax crystal morphology of the leaf surface. Cuticular wax crystals of 8-week-old wheat formed platelets which were connected to a dense network between the adaxial and abaxial leaf sides, and the size of the wax platelets ranged from 0.8 μm to 1 μm in length and from 0.5 μm to 1.1 μm in height [32]. Nevertheless, a few recent studies suggested that platelet-shaped wax crystals were deposited on the adaxial side of wheat flag leaves and tubular/rod-shaped wax structures were observed on the abaxial surface [18–20]. These observations allow one to infer that the wax crystals of the wheat leaf surface may show developmental differences.

Wheat is one of the most important staple crops in the world. Although the morphology and component of cuticular waxes have been extensively studied in wheat [17, 18, 19, 20, 32, 33], it remains unclear how the cuticular wax component and crystal structure of wheat leaves and spikes change with the increasing wheat plant age. Thus, gas chromatography with flame ionization detection (GC-FID), gas chromatography and mass spectrometry (GC-MS) and SEM were applied to investigate the changes in the cuticular wax composition and the crystal morphology of leaves and spikes in parallel, in order to comprehensively understand the wax developmental patterns in wheat leaf and spike.

## Materials and Methods

### Plant material and growth conditions

Four genotypes of wheat from China were grown under natural environmental conditions in the research field of Northwest A&F University, Yangling, Shaanxi province of China during the 2012–2013 and 2013–2014 wheat-growing seasons. Four wheat varieties were selected to represent a wide range of character combinations including both glossy species (A14 and Jing 2001) and glaucous species (Fanmai 5 and Shanken 99). All varieties had winter growth habit. A total of 30 seeds per family were individually hand-planted in a 2-m row at 25-cm apart. All of wheat plants were of the same age, grown in the same field, and subjected to standard cultivation.

### Leaf and spike sampling

To analyze developmental changes in the wax load, composition and morphology during the leaf developmental cycle, leaves were sampled at 50, 100, 200 and 230 days after seed germination. In general, different growth stages of wheat plants at 50, 100, 200 and 230 days represent seedling stage, wintering stage, heading stage and filling stage, respectively. Leaves at 50 and 100 days were young and not completely unfolded. Therefore, the first leaves were sampled from different plants per replicate. At 200 and 230 days, flag leaves were collected. Spikes were harvested randomly from three individual plants at 1, 3, 5, 7, 9 and 15 days after heading (DAH). Leaf and spike samples were divided into two groups: one group was used for GC-MS and GC-FID analysis, and the other was used for wax morphology analysis.

### Cuticular wax extraction

Leaf and spike samples collected at different development stages were immediately immersed in chloroform after tissues were photographed, and shaken for 1 min at room temperature to extract cuticular waxes. The wax-extracted spikes were dried at 50°C for seven days and weighed. A known amount of *n*-tetracosane (C_24_ alkane) was added as an internal standard. The resulting extracts were filtered and dried completely under a gentle stream of nitrogen gas. The samples containing the internal standard were re-dissolved in chloroform, transferred to a GC autosampler vial, and dried again under a stream of nitrogen gas. Subsequently, the extracted samples were treated with 100 μl bis-N,N-(trimethylsilyl) trifluoroacetamide (BSTFA, Sigma) and 100 μl pyridine (Fluka) for 1 h at 70°C to transform hydroxyl containing compounds into their corresponding trimethylsilyl derivatives. Then, the surplus BSTFA was quickly evaporated under nitrogen gas flow and re-dissolved in 500 μl of chloroform for chemical analysis.

### Chemical analysis of cuticular waxes

Chemical composition of the wax extract was analyzed by a capillary gas chromatograph equipped with a Rxi-5ms column (30 m length, i.d 0.25 mm, film thickness 0.25 μm; Restek, USA) and attached to a mass spectrometer (GCMS-QP2010, Shimadzu, Japan) using helium as the carrier gas. GC was carried out with temperature-programmed on-column injection and oven temperature set at 50°C for 2 min, raised by 20°C min^-1^ to 200°C, held for 2 min at 200°C, raised by 2°C min^-1^ to 320°C, and held for 15 min at 320°C. Individual wax components were identified by comparison of their mass spectra with those of authentic standards and literature data. The quantitative compositions of the mixtures were studied using GC-FID (GC-2010 Plus, Shimadzu, Japan; column 60-m Rtx-1, 0.32-mm i.d., df 0.25 μm; Restek, USA). GC was carried out under the conditions as described above but with nitrogen as the carrier gas. Quantification was based on the FID peak areas and the internal standard (*n*-tetracosane), which was added to the wax samples before GC-FID. The total amount of leaf wax components was expressed per unit of leaf surface area, and the total amount of spike wax components was expressed in μg of wax per g of spike (dry weight). The total leaf blade surface areas were calculated with ImageJ software (http://rsb.info.nih.gov/ij/) by measuring the apparent leaf blade areas in digital images and multiplying by 2. All quantitative data are given as mean values and standard deviations (SD).

### SEM

To examine the differences in wax morphology during various stages of leaf and glume development, fresh leaf blades of four wheat varieties were collected at 50, 100, 200, and 230 days of plant growth, respectively. Glumes were sampled at 1, 3, 5, 7, 9 and 15 DAH. The samples were air-dried for seven days in a desiccator at room temperature and then carefully dissected. 3–5 mm completely dried pieces were attached with double adhesive tape to the aluminium stubs and sputter-coated with gold particles using 90-s bursts from a sputter coater. Coated surfaces were investigated using a Hitachi S4800 SEM at an accelerating voltage of 10 kV and a working distance of 8.4 mm.

## Results

### Changes in the amount and individual composition of leaf surface wax during leaf development

**Fig 1:**
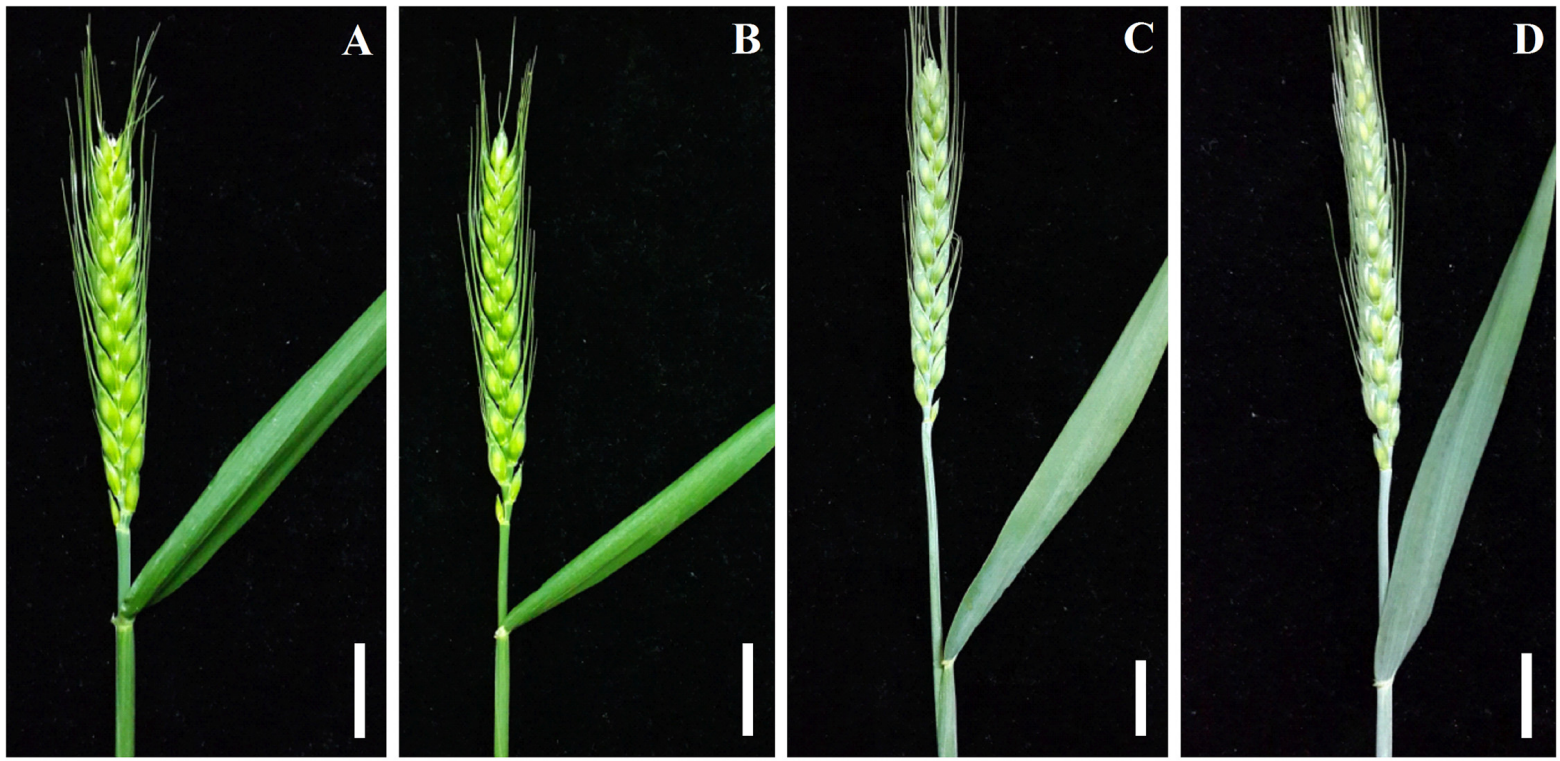
Cuticular wax phenotype on the flag leaf and spike of four wheat varieties. Glossy varieties of A14 (A) and Jing 2001 (B), and glaucous varieties of Fanmai 5 (C) and Shanken 99 (D). The scale bars indicate 2 cm.

**Fig 2:**
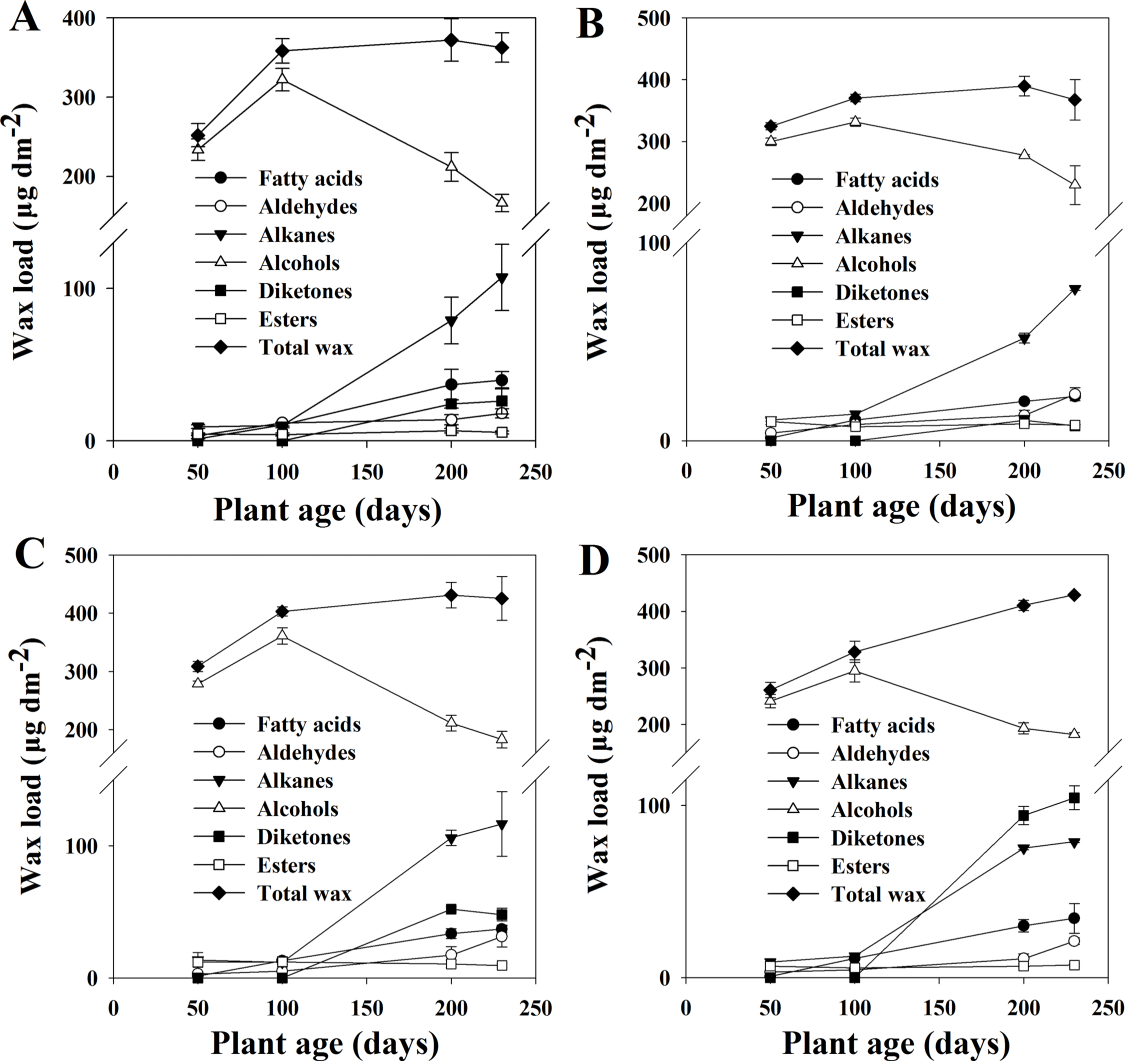
Developmental changes in wax component and the total load on the leaf surface. (A) A14. (B) Jing 2001. (C) Fanmai 5. (D) Shanken 99. Four representative developmental stages (50, 100, 200 and 230 days) were investigated for wax coverage. The absolute amounts of cuticular waxes are expressed as μg dm^-2^ of leaf blade surface area. Each datum point represents a pooled sampled of at least three wheat leaves. Each value represents the mean of three replicates and error bars indicate SD. The amount of diketones is the sum of β- and OH-β-diketones.

The total wax from leaves of the four wheat varieties, including both glossy species (A14 and Jing 2001) and glaucous species (Fanmai 5 and Shanken 99), was extracted with chloroform (Fig 1). The chemical composition of cuticular wax was analyzed at four plant age stages (50, 100, 200 and 230 days). The total leaf wax load of the four wheat varieties increased steadily during initial leaf development between 50 and 200 days, and then decreased from 200 to 230 days, except for Shanken 99, in which the total wax load increased continuously throughout the entire sampling period (Fig 2 and S1 Table). Hence, it can be concluded that a general trend of the total wax load is initially to increase and then decrease during leaf development. For example, the total wax load on Jing 2001 leaves was 324.9, 370.2, 389.4 and 367.4 μg dm^-2^ at 50, 100, 200 and 230 days, respectively (S1 Table).

Between 50 and 100 days of the early stages of leaf development, the cuticular wax composition of leaves was dominated by alcohols (approximately 90%), accompanied by alkanes (2.8–4.4%), esters (1.1–3.9%), aldehydes (1.0–3.3%) and fatty acids (0.2–3.4%). Between 200 and 230 days, the major wax constituents were alcohols (42.5–72.9%), alkanes (13.6–29.4%), and β-diketone (2.0–24.4%), followed by fatty acids (5.2–11.0%), aldehydes (2.7–7.5%) and esters (1.5–2.5%) (S1 Table). The amount of predominant alcohols first increased and reached a maximum (294.6–361.0 μg dm^-2^) at 100 days, and then continuously decreased during further leaf development (Fig 2 and S1 Table). Notably, the relative proportions of alcohols continuously decreased even though the absolute content of alcohols increased between 50 and 100 days. For example, the relative proportions of alcohols on Jing 2001 leaves were 92.3%, 89.5%, 72.9%, and 62.3% at 50, 100, 200, and 230 days, respectively (S1 Table). The most striking change in wax composition was observed with β- and OH-β-diketones. During 50 and 100 days, no β- and OH-β-diketones were detected in wax extracts of wheat leaves. When the leaves were approximately 200 days old, β- and OH-β-diketones occurred for the first time in detectable amounts (10.3–94.1 μg dm^-2^), and then increased or decreased during further leaf development according to different wheat varieties (Fig 2 and S1 Table). In addition, we found that there were large differences in the amounts of β- and OH-β-diketones among different wheat varieties. For example, at 230 days, the amount of β- and OH-β-diketones on glossy variety Jing 2001 was 7.4 μg dm^-2^, but that on glaucous variety Shanken 99 was 104.5 μg dm^-2^ (S1 Table). In contrast, the amounts of alkanes, fatty acids and aldehydes increased steadily from 50 to 230 days (Fig 2 and S1 Table). In general, there were no large changes in the amount of esters during the entire stages of leaf surface development (Fig 2 and S1 Table).

### Chain length distribution of leaf cuticular waxes composition

**Fig 3:**
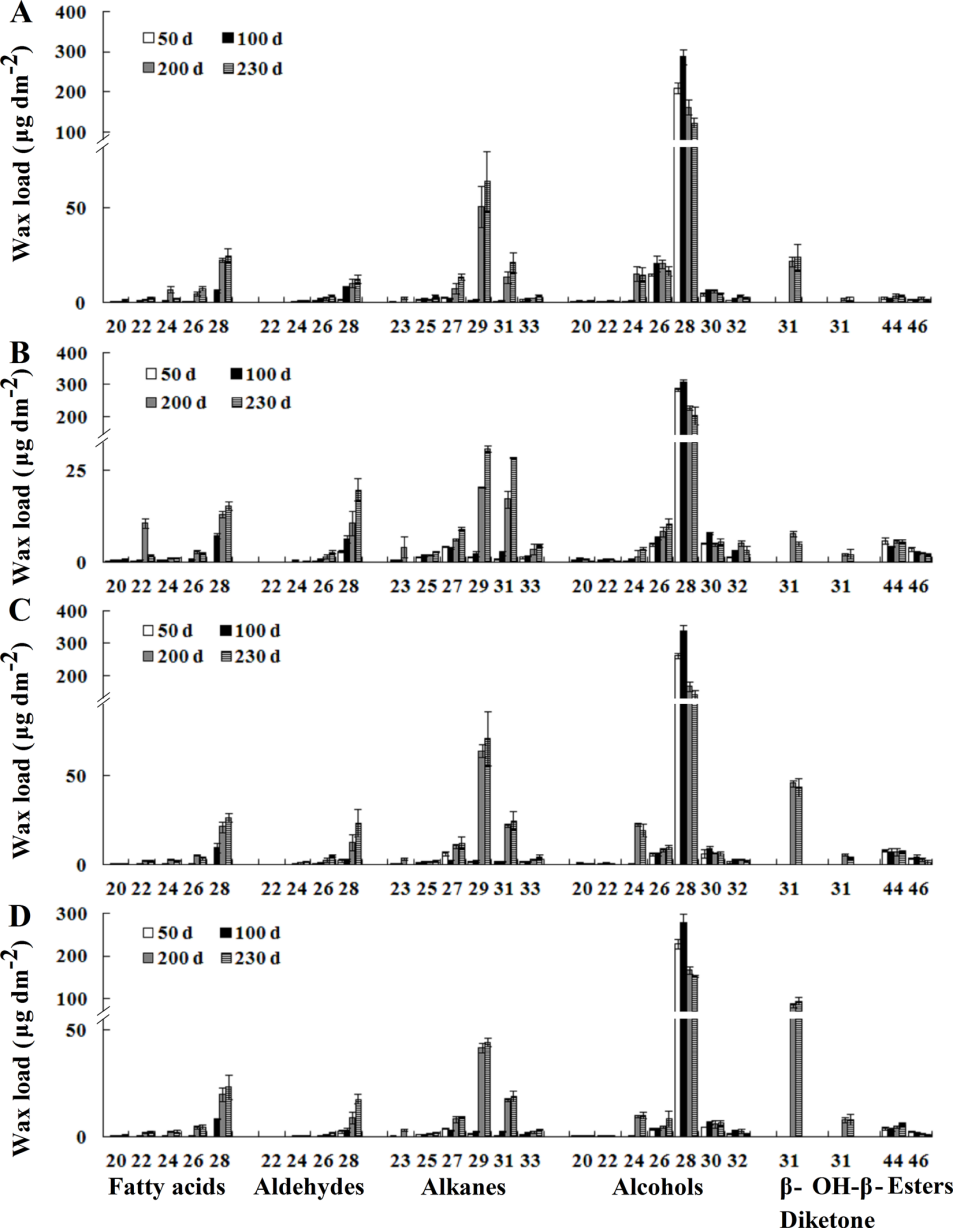
Chain length changes in the individual wax constituent of leaf surface. (A) A14. (B) Jing 2001. (C) Fanmai 5. (D) Shanken 99. Each wax constituent is designated by carbon chain length and is labeled by chemical class along the x-axis. Each value represents the mean of three replicates. Error bars = SD.

The chain length distributions of individual wax constituents were further analyzed by GC-FID and GC-MS. The major alcohols constituent on leaves of the four wheat varieties consisted of even numbers of carbon chain length from C_20_ to C_32_, with C_28_ being the dominant chain length. The alcohols chain length remained constant throughout the growth period. The odd number carbon chain length of alkanes ranged from C_23_ to C_33_ (Fig 3). Interestingly, a shift in the dominant alkanes chain length was observed during leaf development. The dominant alkanes chain length was C_27_ from 50 to 100 days, but C_29_ became the dominant chain length from 200 to 230 days. The aldehydes chain length contained even carbon number chain lengths between C_22_ and C_28_, with a maximum of C_28_. The aldehydes chain length distribution did not change during leaf ontogenesis (Fig 3). Additionally, there was a significant change in the chain length distribution of fatty acids constituent. At 50 days, the chain length distribution ranged from C_20_ to C_24_ or C_20_ to C_26_, without a dominant chain length. However, the chain length distribution became increasingly longer ranging from C_20_ to C_28_ at 100 days, with C_28_ forming the dominant chain length, and then remained constant throughout the remaining period of leaf development. A single carbon chain length, C_31_, was detected for β- and OH-β-diketones. The carbon chain length of esters contained C_44_ to C_46_, with C_44_ being the major chain length (Fig 3). Our studies suggested that the individual wax constituent of the four wheat varieties shared nearly identical changes in chain length distribution during leaf development.

### Morphological changes in leaf surface wax crystals during leaf development

To gain insight into the dynamic development of epicuticular wax crystals, we investigated the wax crystal micromorphology on both the adaxial and abaxial sides of leaf blades by SEM. Leaf blades were obtained from four wheat varieties at four plant ages (50, 100, 200 and 230 days). Based on the classification and terminology of plant epicuticular waxes presented by Barthlott et al [31], we identified two forms of wax crystals on the wheat leaf surface: platelets and tubules (S1–S4 Figs). At 50 days, both the adaxial and abaxial leaf sides of all four wheat varieties were covered with platelet-shaped wax crystals. Some platelets were connected to their neighboring crystals and formed a dense network. The size of the wax platelets was between 0.3 and 0.7 μm in length and between 0.3 and 0.5 μm in height. In general, the wax platelets had irregular margins, which were slightly curved and arranged in widely varying angles towards each other (S1–S4 Figs). SEM showed no significant differences in the wax crystal morphology of leaf blades with different varieties, or between the adaxial and abaxial leaf sides during initial leaf development at 50 days. The crystal shapes at 100 days were similar to that of 50 days, with platelet-shaped structures depositing on both the adaxial and abaxial leaf sides (S1–S4 Figs). Nevertheless, the platelets at 100 days were distinguished by a significantly longer length of 0.6–1.0 μm and a silimar height of 0.3–0.5 μm, compared to those at 50 days. Additionally, the adaxial and abaxial sides of the leaf blades at 100 days showed a crystal structure with more sparse arrangements of plate-shaped wax, suggesting a decrease in the total number of crystalloids present per unit area compared to that at 50 days (S1–S4 Figs). Strikingly, dramatic changes in wax morphology occurred at 200 days. Leaves from glossy species (A14 and Jing 2001) showed significantly different structures with deposits of plate-shaped wax on the adaxial leaf surface and the smooth wax film coverages, without any crystalline structures present, on the abaxial leaf surface (S1 and S2 Figs). In contrast, the epicuticular wax of glaucous species (Fanmai 5 and Shanken 99) formed platelets on adaxial sides of leaf blades and tubule-shaped structures on the abaxial sides of leaf blades. The tubules were approximately 0.1–0.3 μm in diameter and 5–20 μm in length (S3 and S4 Figs). Consequently, it was concluded that the crystals morphology of the abaxial leaf surface changed drastically from 100 to 200 days. The abaxial surface may show a smooth wax film or form tubule-shaped structures at 200 days, depending on the wheat variety. During further leaf development, the cuticular wax layer shared very similar crystals structure between 200 and 230 days (S1–S4 Figs). Taken together, these results clearly show that as plant age increased, epicuticular wax crystals display morphology changes specifically on the abaxial side of the wheat leaf blade.

### Developmental changes in the amount and individual constituent on the spikes of wheat

**Fig 4:**
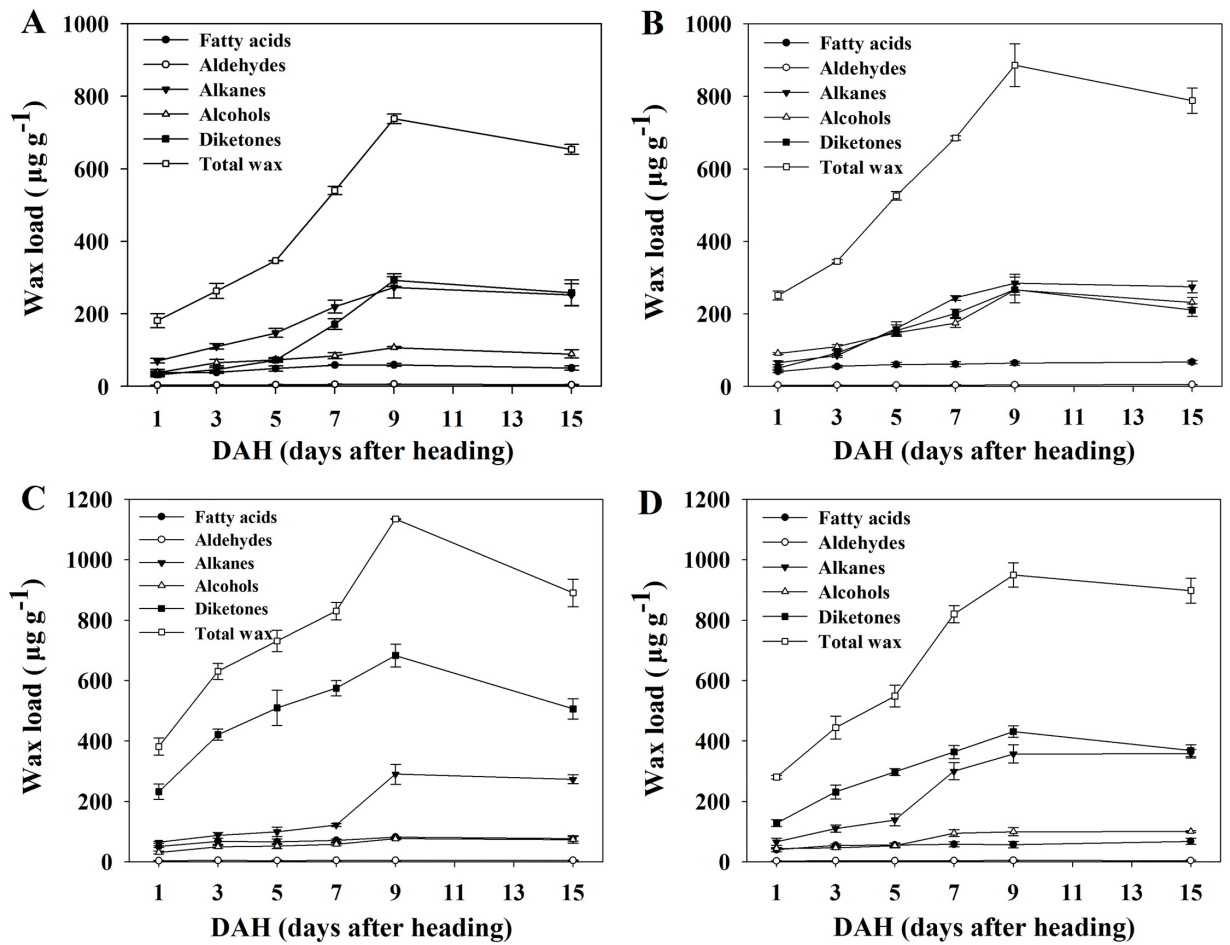
Development changes in the spike surface wax component and the total wax load. (A) A14. (B) Jing 2001. (C) Fanmai 5. (D) Shanken 99. Spike surface wax was measured at six stages (1, 3, 5, 7, 9 and 15 DAH). Wax coverage is expressed as μg g^-1^ of wheat spikes dry weight. Each datum point represents a pooled sampled of at least three spikes. Each value represents the mean of three replicates and error bars indicate SD. The amount of diketones is the sum of β- and OH-β-diketones.

Besides functioning as inflorescence and protectors of the growing grains, spikes play a major role in the production of assimilates for grain filling [34]. To this end, there is no information available on changes of wheat spike wax constituents with different growth stages. Therefore, we investigated the changes in wax amount and composition on spikes of four wheat varieties at six stages (1, 3, 5, 7, 9 and 15 DAH). The total wax load on the spikes of all four wheat varieties increased steadily from 1 to 9 DAH and reached a maximum at 9 DAH, and then decreased from 9 to 15 DAH (Fig 4). For instance, the total wax load on glossy variety Jing 2001 spikes was 250.7, 345.0, 525.6, 685.2, 886.0 and 788.3 μg g^-1^ at 1, 3, 5, 7, 9 and 15 DAH, respectively (S2 Table). For all four wheat varieties, the spikes wax fraction consisted of the five compound classes, including β- and OH-β-diketones, alcohols, alkanes, aldehyde and fatty acids. The wax amount of alkanes, diketones and alcohols constituents continuously increased from 1 to 9 DAH, and then decreased or changed slightly until 15 DAH depending on varieties (Fig 4). During the investigated period of 15 DAH, the wax amount of aldehydes and fatty acids constituents showed a slight fluctuation (Fig 4). These tendencies of changes in spike waxes were shown almost similarly in all four varieties. Studies presented here demonstrated that the total wax amount of wheat spike surface firstly increased and then decreased during spike development.

**Fig 5:**
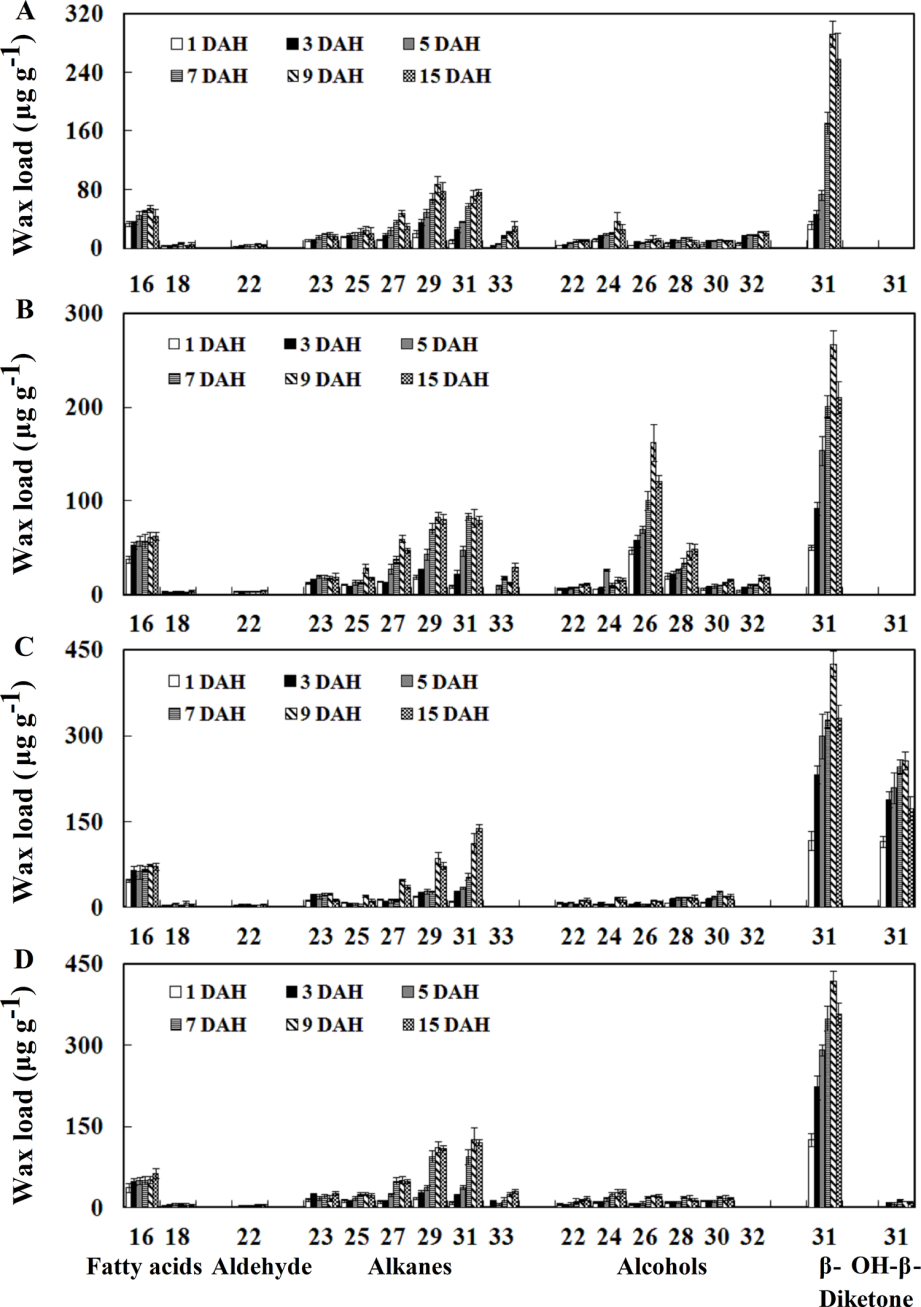
Chain length distribution in the individual wax constituent on spikes. (A) A14. (B) Jing 2001. (C) Fanmai 5. (D) Shanken 99. Each wax constituent is designated by carbon chain length and is labeled by chemical class along the x-axis. Each value represents the mean of three replicates. Error bars = SD.

β- and OH-β-diketones mainly consisted of a single carbon chain length of C_31_. Interestingly, OH-β-diketone was not detected in glossy varieties (Fig 5A and 5B), and a small amount of OH-β-diketone was detected in Shanken 99 (Fig 5D), while Fanmai 5 yielded high amount of OH-β-diketone (Fig 5C), suggesting that the amount of OH-β-diketone strictly depended on varieties. Generally, β- and OH-β-diketones were the major constituent on spikes of glaucous species, with relative portions ranging from 41.0 to 69.6% (S2 Table). The odd number carbon chain length of alkanes ranged from C_23_ to C_31_ or C_23_ to C_33_ depending on wheat varieties, with a relatively sharp maximum at both C_29_ and C_31_. Unlike C_28_ being the dominant chain length in alcohols from leaves, the alcohols constituent of spikes consisted of even numbers of carbon chain length from C_22_ to C_30_ or C_22_ to C_32_, with C_24_ or C_26_ being the dominant chain length. The fatty acids constituent consisted of even numbers of carbon chain length C_16_ and C_18_ with a maximum for C_16_. The aldehyde contained a single even carbon number chain length of C_22_ (Fig 5). Additionally, unlike the chain length changes of leaf surface wax, there were no significant changes in the chain length distributions of all the five wax components on spikes of the four wheat varieties during spike development (Fig 5). In conclusion, our results disclose that the wax amounts and components on the spikes of wheat exhibit the significant developmental changes with increasing spike age.

### Morphological changes in wax crystal on the glumes of wheat

**Fig 6:**
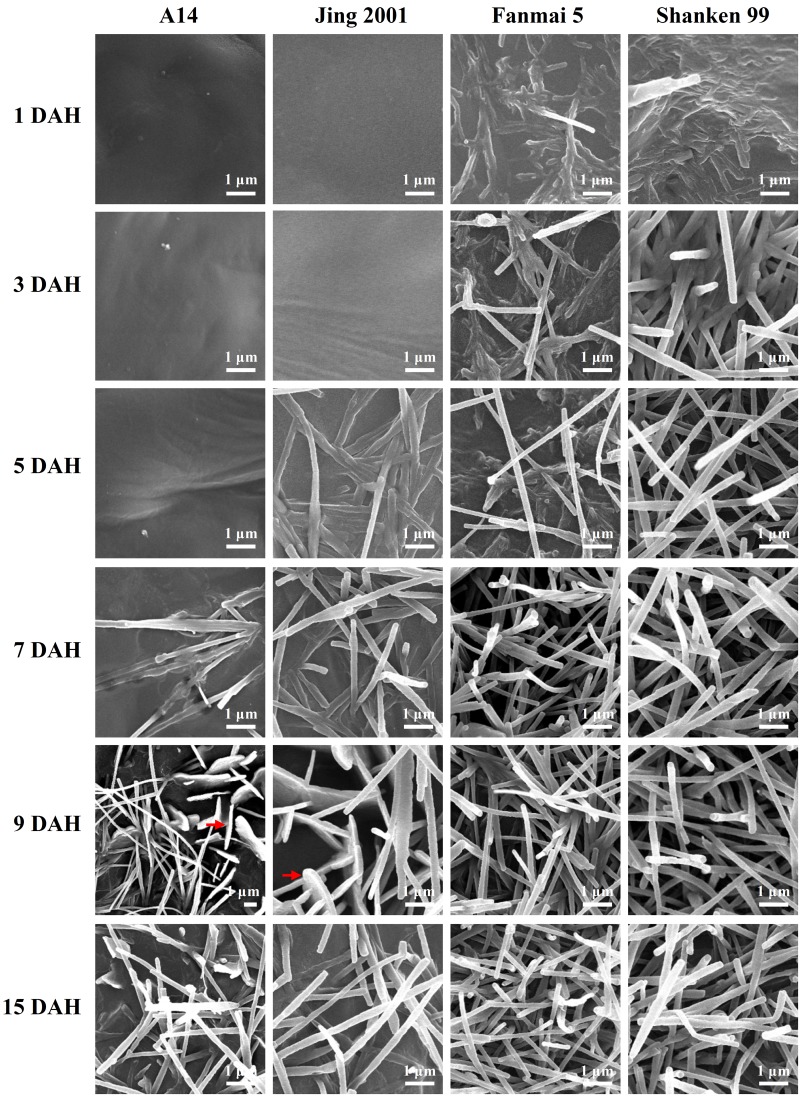
Developmental changes of epicuticular wax crystals on glume surfaces. The six stages of spike development are indicated on the left. The wheat varieties of each column are labeled on the top of the charts. Platelet-shaped wax crystals are indicated by the arrows and the bars indicate 10 μm.

Because the major surface of a spike was covered by glumes, to characterize the morphological development of the spike epicuticular wax crystals, native glume surfaces of four wheat varieties were investigated by SEM at the same six stages. Similarly to the leaves, the glumes of wheat were also covered with two forms of crystalloids: platelets as well as tubules. Unexpectedly, the platelets were only present on glossy species, whereas tubules were present on all four varieties (Fig 6). The glume wax crystals developed differently between glossy and glaucous varieties. For glossy species, at 1 DAH, the glume surfaces were covered by a relatively smooth film without any crystal structures present, typically rendering their surfaces glossy. This trend continued from 1 to 3 DAH. At 5 DAH, glumes of A14 still appeared smooth. In contrast, a small amount of tubules began to deposit on the glumes of Jing 2001. At 7 DAH, tubules were recognized for the first time on A14 glumes, and Jing 2001 glumes formed a more dense covering of tubules (Fig 6). Strikingly, at 9 DAH, both platelets and tubules can be found on glumes of A14 and Jing 2001. Notwithstanding, during spike development, platelets disappeared with only tubules present on the glume surfaces of glossy species at 15 DAH, suggesting a very short window to view the plate-shaped wax crystals on glume (Fig 6). For glaucous species, a small amount of tubules began to deposit at 1 DAH, and tubule-shaped wax crystals became considerably great density during the glume development. This trend continued until 15 DAH. At 15 DAH, the glumes of all four wheat varieties showed the tubule-shaped wax structure, and the tubules were parallel to the plane of the glume surface, with a diameter of 0.1–0.3 μm and a length of 5–20 μm (Fig 6). Compared to glossy species, glaucous species displayed a dense array of tubules from 1 to 15 DAH (Fig 6). Consequently, it was concluded that tubule-shaped wax crystals first appeared, and platelets started to form, finally, platelets disappeared with only tubules present on the glume surface of glossy species. In contrast, only tubules were present on glaucous species throughout the entire period of 15 DAH (Fig 6). Taken together, these results again demonstrate that the development of epicuticular wax crystals is a dynamic process and that platelets and tubules on wheat glume surfaces can be formed rapidly within a few days.

## Discussion

Despite several reports on wheat wax content and morphology [17, 18, 19, 20, 32, 33], this is the first detailed and precise characterization of the developmental changes in cuticular wax composition and morphology on the leaves and spikes during wheat plant growth. Wheat leaves vary in size according to growth stage. We found that the total amounts of cuticular wax on leaf surfaces increased continuously from 50 to 200 days, matching the time period of leaf expansion, and then decreased until the end of leaf development, except for Shanken 99, in which wax levels gradually increased throughout the analyzed leaf development stages (Fig 2). We speculate that Shanken 99 leaves continue to expand at 230 days and that the total wax amounts may decrease during further leaf development. Therefore, it can be concluded that wax biosynthesis is in full progress during early leaf development. However, when leaf expansion is complete, wax accumulation ceases. The wax amount of all four wheat varieties constantly changed during the investigated period of 230 days (Fig 2 and S1 Table). This result indicates that any amount of wax given for wheat in previous report is only representative of a certain plant age [17, 18, 26, 32, 35]. Additionally, it should be noted that alcohols are always the major components of cuticular waxes mixture throughout leaf development. When β- and OH-β-diketones presented, the alcohol content decreased correspondingly.

In the present study, we also investigated the chain length changes in individual wax constituents of leaves. Generally, these results are in accordance with observations on other wheat cultivars [17, 35]. Interestingly, there was a significant change in the alkane chain length distribution, in which the dominant chain length of alkanes shifted from C_27_ to C_29_ during leaf development. Likewise, the chain length for fatty acids became increasingly longer from 50 to 100 days, and then remained almost constant in chain length distribution between 100 and 230 days (Fig 3). Similar to developmental changes of leaf surfaces wax, the amount and composition of spike surfaces wax showed the dynamic changes. The total spike wax load first increased, and then decreased during spike development. The amount of alkanes, diketones and alcohols continuously changed throughout the entire period of spike growth. Compared to leaves, alkanes and diketones were major factors that caused the waxes changes in spike growth. There was no significant change in the chain length distributions of all the five wax components of spikes.

The prevailing consensus of opinion regarding the diversity of wax crystals on plant surfaces is that the various morphologies result from self-assembly processes, based mainly on the chemical composition of the waxes [32]. Our results showed that epicuticular wax crystals patterns on wheat leaf surface consisted of platelets and tubules, and the crystals morphology and size changed constantly during leaf development. Between 50 and 100 days of plant, both the adaxial and abaxial sides of leaf blades were covered by platelet-shaped wax crystals (S1–S4 Figs). Similar to our results, the cuticular wax on both sides of the blades from 8-week-old *T*. *aestivum* plants formed platelets [32]. Interestingly, from 200 to 230 days, the adaxial wax layer was characterized by a homogeneous coverage of platelet-shaped crystals, but the abaxial side of flag leaf blade changed to a smooth wax film in a glossy varieties or tubules in a glaucous varieties. Previous studies have shown that the adaxial wax bloom in wheat leaves consisted mainly of deposits of thin wax plates, and fibrillar waxes predominated on the abaxial surface of flag leaves [18, 20, 36]. For spikes, this is no literature report on the developmental changes in crystal morphology on wheat glumes. Our results showed that epicuticular wax of glumes was composed of platelets and tubules. Interestingly, for glossy species, tubules first formed, and then platelets began to appear, finally, platelets disappeared with only tubules depositing. Nevertheless, for glaucous species, only tubules appeared during the entire sampling period of 15 DAH. In particular, platelets and tubules on glume surfaces can be formed rapidly within a few days. Based on these results of the chemical composition and the crystals morphology, it is reasonable that the wax chemical composition changes in different stages correspondingly cause the wax crystals changes.

Epicuticular wax layer that covers leaves, fruits and stems, gives the plant surface a glaucous or gray appearance [22, 29, 30]. It has been known that platelet crystals are positively correlated with alcohols on the wheat leaf surface and the recrystallization of the pure octacosan-1-ol constituent of wheat waxes formed upright platelets [32]. Prior studies have demonstrated that tubules are dominated by secondary alcohol (nonacosan-10-ol) or β-diketone [37–39]. Barthlott et al also proposed the term ‘β-diketone tubules’ [40]. The β-diketone tubules mainly contribute to the glaucous phenotype in wheat [17, 18, 19, 41]. In this study, glaucous and glossy wheat varieties were used for wax analysis. Our results showed that the abaxial leaf surface of glossy varieties displayed a smooth wax film between 200 and 230 days (S1 and S2 Figs), while glaucous varieties were covered with wax tubules (S3 and S4 Figs). The wax chemical data indicated that β- and OH-β-diketones could be detected in both glossy and glaucous leaves. This result suggested that the amount of β- and OH-β-diketones must reach a certain threshold before tubules could be observed. For example, when the amounts of β- and OH-β-diketones ranged from 24.1 to 26.1 μg dm^-2^, tubules were absent on the abaxial surface of A14. When the amounts of β- and OH-β-diketones ranged from 48.0 to 52.1 μg dm^-2^, tubule-shaped wax crystalloids are present on the abaxial surface of the Fanmai 5 leaf, suggesting that 48.0 μg dm^-2^ of β- and OH-β-diketones is a critical threshold value for the occurrence of tubules, at least in wheat leaf. Our results also showed that β-diketone could be detected in the spikes of both glossy and glaucous varieties, but OH-β-diketone could be detected only in the spikes of glaucous varieties. Consequently, it was concluded that β- and OH-β-diketones could be synthesized by different enzymes and these varieties were good materials for further research on the biosynthesis of OH-β-diketone.

## Conclusions

Our results clearly indicated that the cuticular wax on wheat leaves and spikes represented a highly dynamic system, and the individual wax component and wax crystal gradually changed during the investigated season. The general accumulation trend of the total wax amount of wheat leaf and spike surfaces is first to increase and then decrease during plant development, but these changes were caused by different compound classes between leaf and spike. Alcohols are the predominant wax constituent on leaves, but the relative percentages decrease continuously throughout the entire period of leaf growth. β- and OH-β-diketones are abundant in spikes, particularly in glaucous varieties. There was a significant shift in the chain length distribution of alkanes between early stage leaf and flag leaf. Unlike C_28_ alcohol being the dominant chain length in leaf waxes, the dominant alcohol chain length of spikes became C_24_ or C_26_ depending on varieties. Epicuticular wax crystals on wheat leaf and glume surfaces consist of platelets and tubules, and the crystal structures change constantly throughout plant growth. In particular, platelets and tubules on glume surfaces could be formed rapidly within a few days.

## Supporting Information

S1 Fig Epicuticular wax crystals patterns on both the adaxial and abaxial leaf surfaces of the wheat variety A14 detected by SEM at four stages of plant development. The four stages of plant development are indicated on the left. The adaxial and abaxial leaf side and the magnification of each column are labeled on the top. The micrographs are at a resolution of 10 000× and 30 000×, and the bars indicate 1 μm and 0.3 μm, respectively.
(TIF)

S2 Fig Epicuticular wax crystals on both the adaxial and abaxial leaf surfaces of Jing 2001. The four stages of plant development are indicated on the left. The adaxial and abaxial leaf side and the magnification of each column are labeled on the top. The micrographs are at a resolution of 10 000× and 30 000×, and the bars indicate 1 μm and 0.3 μm, respectively.
(TIF)

S3 Fig Epicuticular wax crystals on both the adaxial and abaxial leaf surfaces of Fanmai 5. The four stages of plant development are indicated on the left. The adaxial and abaxial leaf sides and the magnification of each column are labeled on the top. The micrographs are at a resolution of 10 000× and 30 000×, and the bars indicate 1 μm and 0.3 μm, respectively.
(TIF)

S4 Fig Epicuticular wax crystals patterns on both the adaxial and abaxial leaf surfaces of the wheat variety Shanken 99 at four stages of plant development. The four stages of plant development are indicated on the left. The adaxial and abaxial leaf sides and the magnification of each column are labeled on the top of the charts. The micrographs are at a resolution of 10 000× and 30 000×, and the bars indicate 1 μm and 0.3 μm, respectively.
(TIF)

S1 Table Cuticular wax compositions on leaves of glossy varieties (A14 and Jing 2001) and glaucous varieties (Fanmai 5 and Shanken 99) at 50, 100, 200 and 230 days of plant development. (DOC)

S2 Table Cuticular wax compositions on spikes of wheat varieties A14, Jing 2001, Fanmai 5 and Shanken 99 at 1, 3, 5, 7, 9 and 15 DAH. (DOC)
