# Correction: Developmental Changes in Composition and Morphology of Cuticular Waxes on Leaves and Spikes of Glossy and Glaucous Wheat (*Triticum aestivum* L.)

**DOI:** 10.1371/journal.pone.0143671

**Published:** 2015-11-18

**Authors:** The PLOS ONE Staff

## Notice of Republication

Figs 1–6 were published in error. The publisher apologizes for the error. The error was corrected in the HTML and PDF versions of this article on November 9, 2015, and the corrected figures are available in the supporting information of this article.

## Supporting Information

S1 File Correct Figs 1–6. (ZIP)
