# Supplementary figures and images for: Developmental Changes in Composition and Morphology of Cuticular Waxes on Leaves and Spikes of Glossy and Glaucous Wheat (Triticum aestivum L.)
Source: PLoS One. 2015 Oct 27;10(10):e0141239. doi: 10.1371/journal.pone.0141239 (PMC4624236; doi:10.1371/journal.pone.0141239)

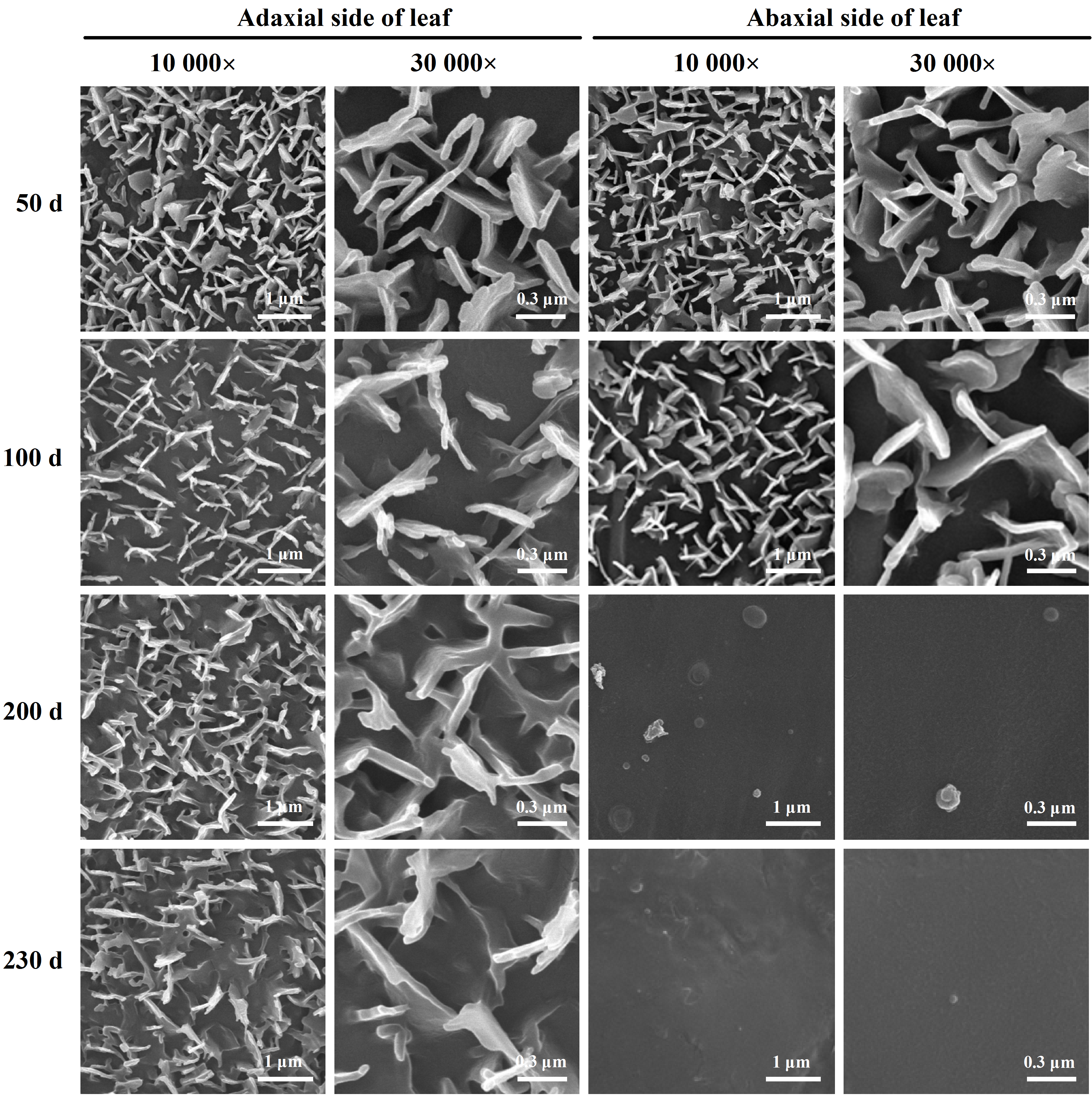

Supplement: S1 Fig — The four stages of plant development are indicated on the left. The adaxial and abaxial leaf side and the magnification of each column are labeled on the top. The micrographs are at a resolution of 10 000× and 30 000×, and the bars indicate 1 μm and 0.3 μm, respectively. (TIF) [file pone.0141239.s001.tif]

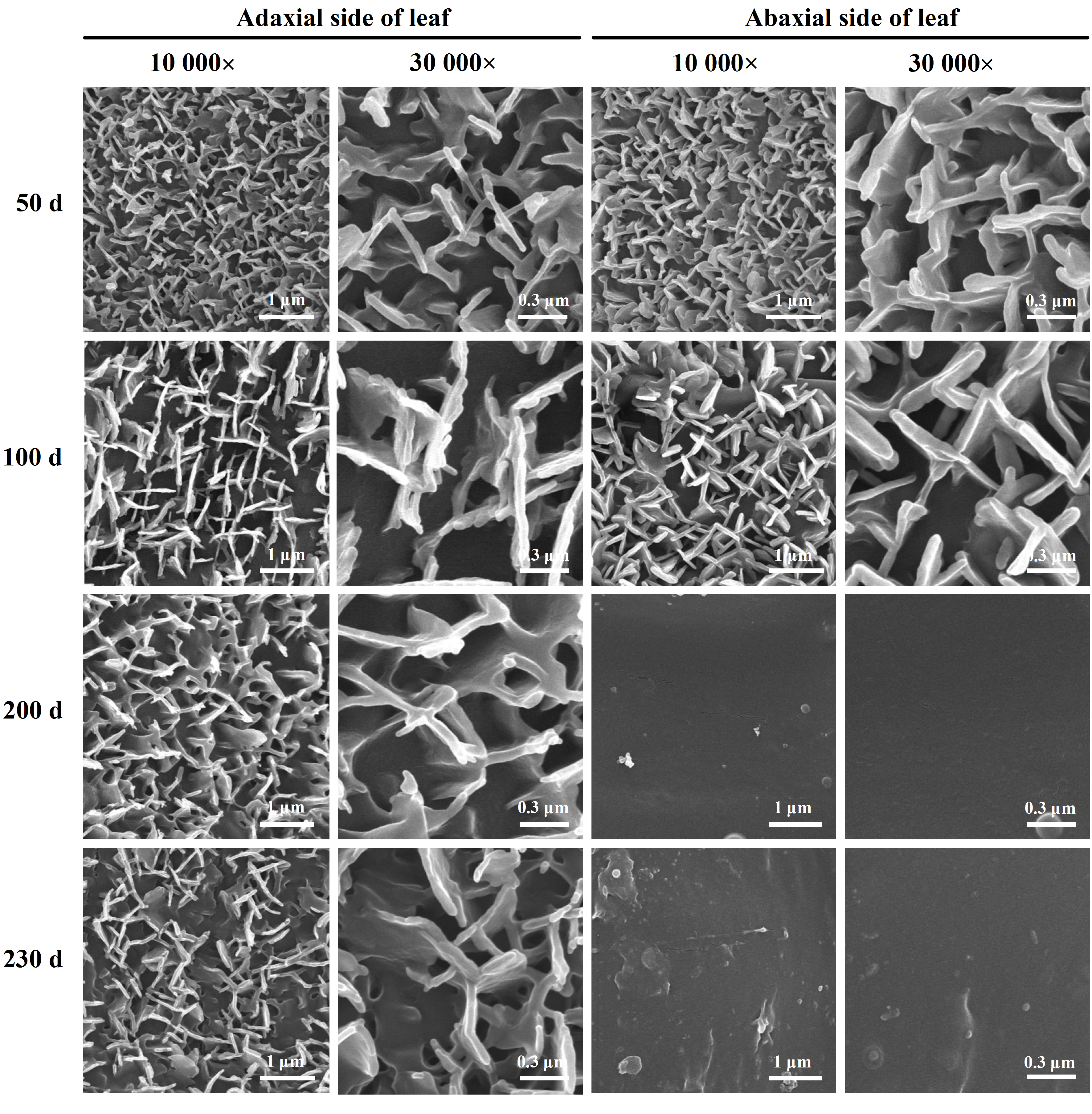

Supplement: S2 Fig — The four stages of plant development are indicated on the left. The adaxial and abaxial leaf side and the magnification of each column are labeled on the top. The micrographs are at a resolution of 10 000× and 30 000×, and the bars indicate 1 μm and 0.3 μm, respectively. (TIF) [file pone.0141239.s002.tif]

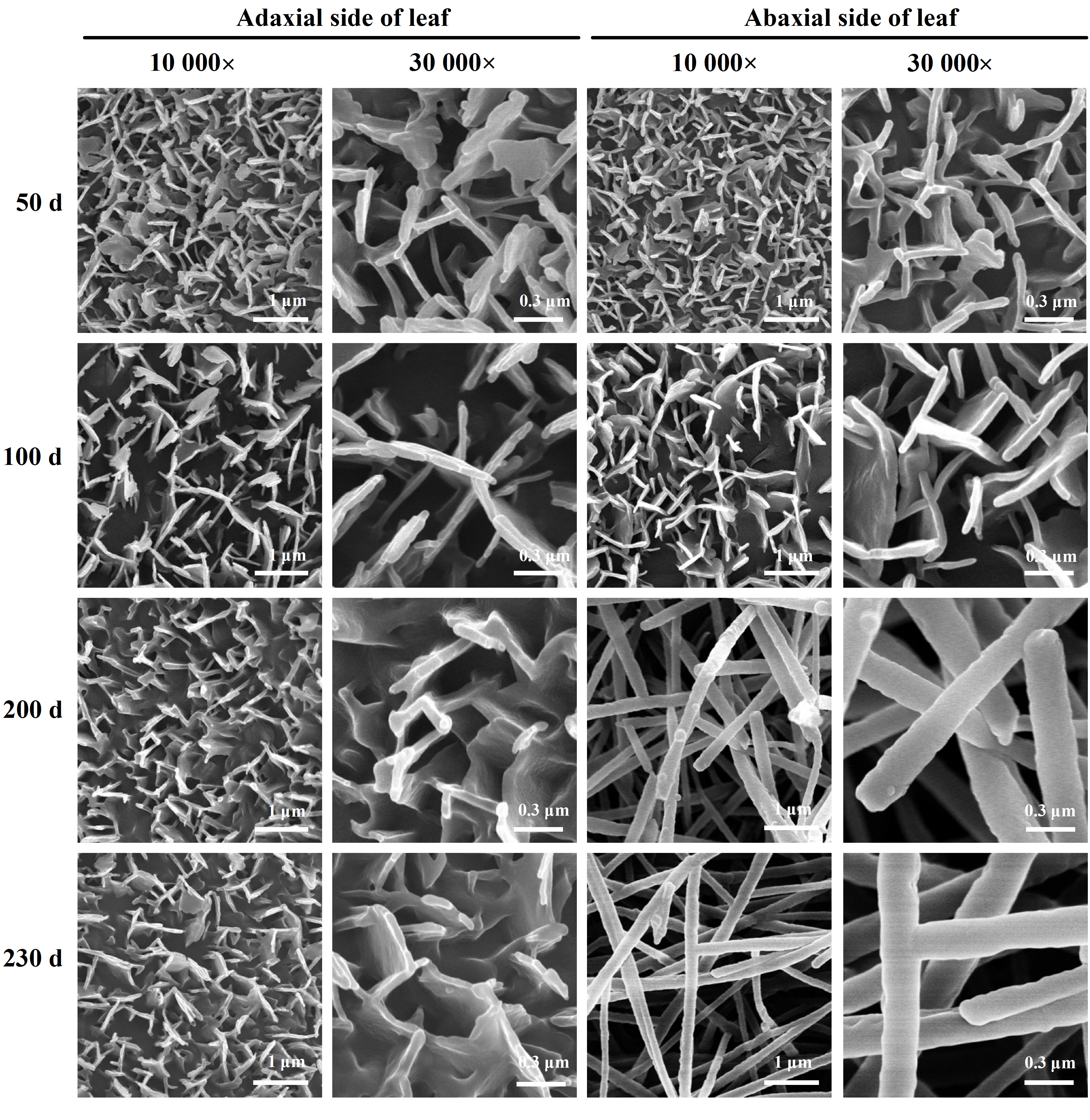

Supplement: S3 Fig — The four stages of plant development are indicated on the left. The adaxial and abaxial leaf sides and the magnification of each column are labeled on the top. The micrographs are at a resolution of 10 000× and 30 000×, and the bars indicate 1 μm and 0.3 μm, respectively. (TIF) [file pone.0141239.s003.tif]

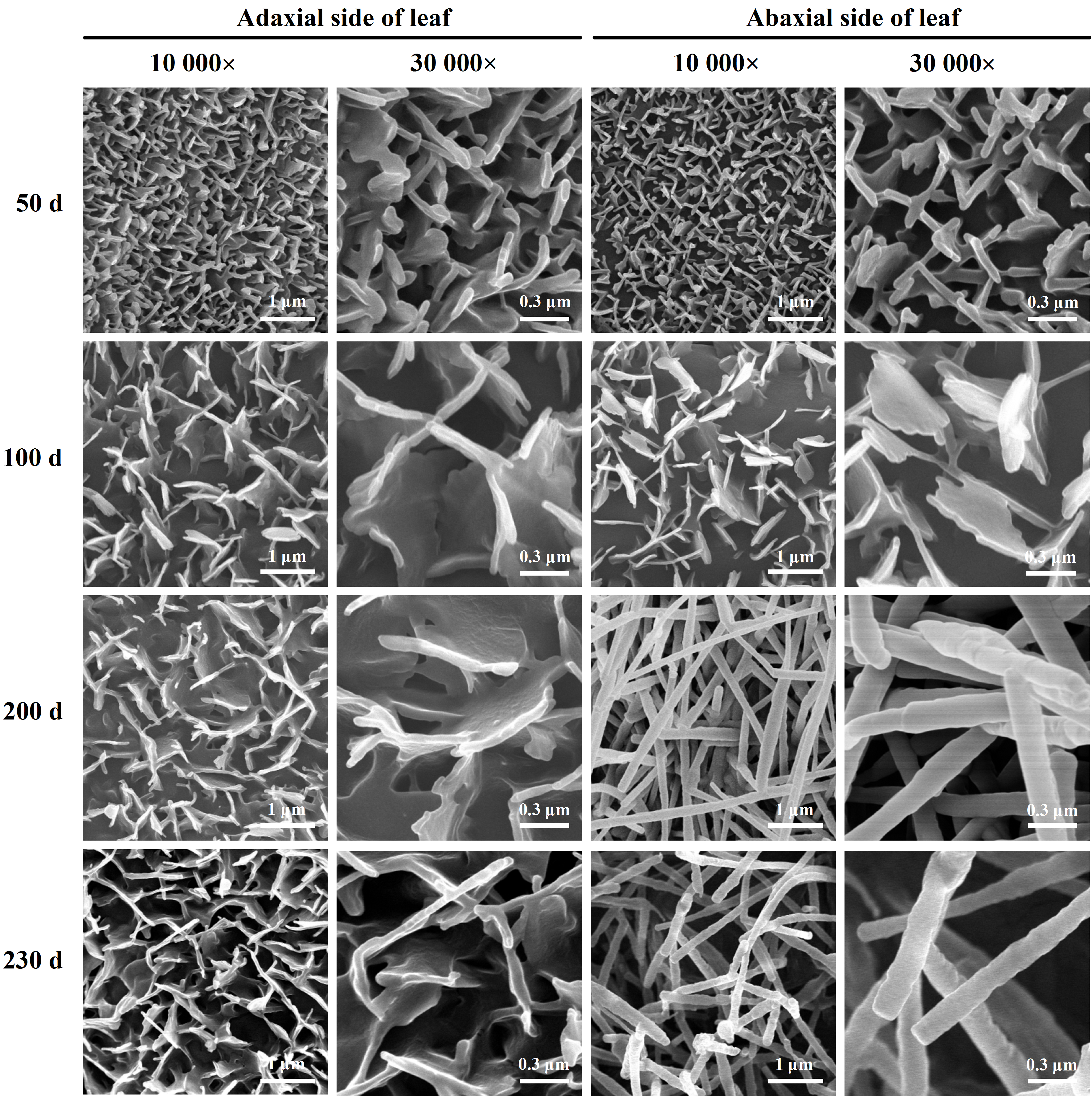

Supplement: S4 Fig — The four stages of plant development are indicated on the left. The adaxial and abaxial leaf sides and the magnification of each column are labeled on the top of the charts. The micrographs are at a resolution of 10 000× and 30 000×, and the bars indicate 1 μm and 0.3 μm, respectively. (TIF) [file pone.0141239.s004.tif]
