# Supplementary material for: Developmental Changes in Composition and Morphology of Cuticular Waxes on Leaves and Spikes of Glossy and Glaucous Wheat (Triticum aestivum L.)
Source: PLoS One. 2015 Oct 27;10(10):e0141239. doi: 10.1371/journal.pone.0141239 (PMC4624236; doi:10.1371/journal.pone.0141239)
Supplement: S1 Table — (DOC) [file pone.0141239.s005.doc]

**S1 Table.** Cuticular wax compositions on leaves of glossy cultivars (A14 and Jing 2001) and glaucous cultivars (Fanmai 5 and Shanken 99) at 50, 100, 200 and 230 days of plant development

| Variety | Plant age | Measurement | Fatty acids | Aldehydes | Alkanes | Alcohols | β- and OH-β -Diketones | Esters | Total load |
| --- | --- | --- | --- | --- | --- | --- | --- | --- | --- |
| A14 | 50 d | Amount | 1.2 ± 0.1 | 3.4 ± 0.1 | 9.2 ± 1.2 | 233.5 ± 13.7 | ND | 4.5 ± 0.6 | 251.8 ± 14.6 |
|  |  | Percentage | 0.5 ± 0.1 | 1.3 ± 0.1 | 3.6 ± 0.4 | 92.7 ± 0.6 | ND | 1.8 ± 0.2 | 100 |
|  | 100 d | Amount | 10.6 ± 1.0 | 11.7 ± 0.4 | 9.9 ± 1.5 | 321.9 ± 14.1 | ND | 4.1 ± 0.9 | 358.2 ± 15.3 |
|  |  | Percentage | 2.9 ± 0.2 | 3.3 ± 0.3 | 2.8 ± 0.3 | 89.9 ± 0.7 | ND | 1.1 ± 0.2 | 100 |
|  | 200 d | Amount | 36.9 ±10.0 | 13.8 ± 3.3 | 78.9 ± 15.2 | 211.7 ± 18.1 | 24.1 ± 2.7 | 6.5 ± 1.4 | 371.9 ± 26.9 |
|  |  | Percentage | 10.0 ± 2.8 | 3.7 ± 0.8 | 21.2 ± 3.2 | 56.9 ± 0.8 | 6.5 ± 1.2 | 1.8 ± 0.4 | 100 |
|  | 230 d | Amount | 39.7 ± 5.6 | 18.0 ± 3.0 | 107.2 ± 21.8 | 166.2 ± 11.1 | 26.1 ± 8.6 | 5.4 ± 0.9 | 362.6 ± 18.6 |
|  |  | Percentage | 11.0 ± 1.6 | 5.0 ± 1.1 | 29.4 ± 4.8 | 45.9 ± 4.1 | 7.2 ± 2.3 | 1.5 ± 0.3 | 100 |
| Jing 2001 | 50 d | Amount | 1.3 ± 0.3 | 3.8 ± 0.5 | 10.4 ± 0.7 | 299.8 ± 6.1 | ND | 9.6 ± 1.5 | 324.9 ± 5.8 |
|  |  | Percentage | 0.4 ± 0.1 | 1.2 ± 0.1 | 3.2 ± 0.2 | 92.3 ± 0.2 | ND | 3.0 ± 0.5 | 100 |
|  | 100 d | Amount | 10.4 ± 0.7 | 8.1 ± 1.2 | 13.4 ± 1.5 | 331.3 ± 6.6 | ND | 7.0 ± 0.4 | 370.2±6.1 |
|  |  | Percentage | 2.8 ± 0.2 | 2.2 ± 0.3 | 3.6 ± 0.5 | 89.5 ± 0.4 | ND | 1.9 ± 0.1 | 100 |
|  | 200 d | Amount | 19.7 ± 0.7 | 12.7 ± 2.7 | 51.8 ± 2.5 | 277.4 ± 1.7 | 10.3 ± 0.7 | 8.5 ± 0.5 | 389.4 ± 15.8 |
|  |  | Percentage | 5.2 ± 0.2 | 3.3 ± 0.7 | 13.6 ± 0.8 | 72.9 ± 1.7 | 2.7 ± 0.2 | 2.2 ± 0.2 | 100 |
|  | 230 d | Amount | 22.3 ± 0.8 | 23.5 ± 3.3 | 76.7 ± 0.9 | 229.7 ± 31.3 | 7.4 ± 0.8 | 7.8 ± 0.4 | 367.4 ± 32.8 |
|  |  | Percentage | 6.1 ± 0.5 | 6.4 ± 0.9 | 21.0 ± 2.0 | 62.3 ± 3.1 | 2.0 ± 0.2 | 2.1 ± 0.2 | 100 |
| Fanmai 5 | 50 d | Amount | 1.4 ± 0.5 | 3.0 ± 0.8 | 13.6 ± 5.6 | 278.8 ± 5.1 | ND | 11.9 ± 0.9 | 308.7 ± 8.8 |
|  |  | Percentage | 0.4 ± 0.1 | 1.0 ± 0.3 | 4.4 ± 1.7 | 90.3 ± 1.8 | ND | 3.9 ± 0.4 | 100 |
|  | 100 d | Amount | 13.1 ± 3.0 | 5.1 ± 0.8 | 11.8 ± 1.5 | 361.0 ± 13.9 | ND | 12.1 ± 3.6 | 403.1 ± 8.0 |
|  |  | Percentage | 3.3 ± 0.8 | 1.3 ± 0.2 | 2.9 ± 0.4 | 89.5 ± 1.7 | ND | 3.0 ± 0.9 | 100 |
|  | 200 d | Amount | 33.6 ± 3.6 | 17.4 ± 6.4 | 106.1 ± 5.7 | 211.6 ± 13.7 | 52.1 ± 1.9 | 10.6 ± 2.6 | 431.2 ± 21.8 |
|  |  | Percentage | 7.8 ± 0.6 | 4.0 ± 1.4 | 24.6 ± 0.6 | 49.0 ± 1.7 | 12.1 ± 0.5 | 2.5 ± 0.6 | 100 |
|  | 230 d | Amount | 37.1 ± 2.8 | 31.3 ± 7.9 | 116.6 ± 24.5 | 183.1 ± 14.3 | 48.0 ± 4.8 | 9.3 ± 1.6 | 425.3 ± 37.5 |
|  |  | Percentage | 8.8 ± 0.9 | 7.5 ± 2.5 | 27.2 ± 3.7 | 43.1 ± 0.5 | 11.3 ± 0.3 | 2.2 ± 0.3 | 100 |
| Shanken 99 | 50 d | Amount | 0.6 ± 0.2 | 3.2 ± 0.7 | 9.2 ± 0.6 | 241.4 ± 11.7 | ND | 6.7 ± 0.7 | 261.0 ± 13.6 |
|  |  | Percentage | 0.2 ± 0.1 | 1.2 ± 0.2 | 3.5 ± 0.1 | 92.5 ± 0.4 | ND | 2.6 ± 0.1 | 100 |
|  | 100 d | Amount | 11.2 ± 0.6 | 4.5 ± 0.8 | 12.5 ± 0.8 | 294.6 ± 19.3 | ND | 5.6 ± 0.9 | 328.4 ± 18.7 |
|  |  | Percentage | 3.4 ± 0.1 | 1.4 ± 0.3 | 3.8 ± 0.2 | 89.7 ± 0.8 | ND | 1.7 ± 0.4 | 100 |
|  | 200 d | Amount | 30.1 ± 3.6 | 11.0 ± 2.9 | 75.2 ± 0.8 | 193.3 ± 10.0 | 94.1 ± 5.3 | 6.7 ± 0.4 | 410.4 ± 9.1 |
|  |  | Percentage | 7.3 ± 0.9 | 2.7 ± 0.6 | 18.3 ± 0.2 | 47.1 ± 1.6 | 23.0 ± 1.5 | 1.6 ± 0.1 | 100 |
|  | 230 d | Amount | 34.4 ± 8.6 | 21.2 ± 1.9 | 79.0 ± 0.6 | 182.5 ± 2.9 | 104.5 ± 6.9 | 7.3 ± 0.9 | 428.8 ± 2.7 |
|  |  | Percentage | 8.0 ± 2.0 | 4.9 ± 0.4 | 18.4 ± 0.1 | 42.5 ± 0.8 | 24.4 ± 1.8 | 1.7 ± 0.2 | 100 |

Mean values (μg dm^-2^) of total wax loads, amount and percentage (%) of individual compound classes are given with SD (*n* = 3). ND, not detected.
