# Supplementary material for: Developmental Changes in Composition and Morphology of Cuticular Waxes on Leaves and Spikes of Glossy and Glaucous Wheat (Triticum aestivum L.)
Source: PLoS One. 2015 Oct 27;10(10):e0141239. doi: 10.1371/journal.pone.0141239 (PMC4624236; doi:10.1371/journal.pone.0141239)
Supplement: S2 Table — (DOC) [file pone.0141239.s006.doc]

**S2 Table. Cuticular wax compositions on spikes of wheat cultivars A14, Jing 2001, Fanmai 5 and Shanken 99 at 1, 3, 5, 7, 9 and 15 DAH**

| Variety | DAH | Measurement | Fatty acids | Aldehydes | Alkanes | Alcohols | β- and OH-β -Diketones | Total load |
| --- | --- | --- | --- | --- | --- | --- | --- | --- |
| A14 | 1 | Amount | 37.9 ±3.8 | 2.9 ± 0.7 | 70.2 ± 6.5 | 38.1 ± 8.2 | 31.8 ± 5.9 | 181.0 ± 19.1 |
|  |  | Percentage | 21.0 ± 2.4 | 1.6 ± 0.4 | 38.8 ± 0.5 | 20.9 ± 2.2 | 17.6 ± 2.8 | 100 |
|  | 3 | Amount | 38.6 ± 2.6 | 3.2 ± 0.9 | 109.8 ± 7.7 | 65.3 ± 9.0 | 46.1 ± 5.7 | 263.0 ± 20.9 |
|  |  | Percentage | 14.7 ± 0.5 | 1.2 ± 0.3 | 41.8 ± 1.2 | 24.8 ± 1.7 | 17.6 ± 2.0 | 100 |
|  | 5 | Amount | 49.1 ± 6.9 | 4.4 ± 1.4 | 147.1 ± 12.0 | 73.3 ± 3.2 | 72.7 ± 6.5 | 346.5 ± 0.4 |
|  |  | Percentage | 14.2 ± 2.0 | 1.3 ± 0.4 | 42.4 ± 3.4 | 21.2 ± 0.9 | 21.0 ± 1.9 | 100 |
|  | 7 | Amount | 58.8 ± 0.3 | 5.8 ± 0.4 | 219.6 ± 17.6 | 84.2 ± 8.6 | 171.5 ± 15.0 | 539.9 ± 11.4 |
|  |  | Percentage | 10.9 ± 0.3 | 1.1 ± 0.1 | 40.6 ± 2.4 | 15.6 ± 1.3 | 31.8 ± 3.4 | 100 |
|  | 9 | Amount | 59.1 ± 4.4 | 6.3 ± 0.3 | 273.1 ± 29.3 | 107.0 ± 2.6 | 292.5 ± 17.6 | 738.0 ± 13.3 |
|  |  | Percentage | 8.0 ± 0.5 | 0.8 ± 0.1 | 37.0 ± 3.3 | 14.5 ± 0.6 | 39.6 ± 3.1 | 100 |
|  | 15 | Amount | 50.2 ± 5.8 | 4.2 ± 0.9 | 252.0 ± 30.6 | 89.1 ± 11.7 | 258.3 ± 35.4 | 653.7 ± 13.6 |
|  |  | Percentage | 7.7 ± 0.7 | 0.6 ± 0.1 | 38.5 ± 3.9 | 13.6 ± 1.5 | 39.6 ± 6.2 | 100 |
| Jing 2001 | 1 | Amount | 40.7 ± 2.8 | 3.4 ± 0.3 | 65.4 ± 5.0 | 90.8 ± 2.5 | 50.4 ± 2.4 | 250.7 ± 12.3 |
|  |  | Percentage | 16.2 ± 0.4 | 1.4 ± 0.2 | 26.1 ± 0.8 | 36.2 ± 0.8 | 20.1 ± 0.2 | 100 |
|  | 3 | Amount | 55.0 ± 3.6 | 3.5 ± 0.9 | 85.4 ± 5.3 | 109.1 ± 6.1 | 92.0 ± 7.0 | 345.0 ± 4.5 |
|  |  | Percentage | 15.9 ± 0.9 | 1.0 ± 0.3 | 24.7 ± 1.5 | 31.6 ± 1.6 | 26.7 ± 2.1 | 100 |
|  | 5 | Amount | 60.2 ± 6.0 | 3.8 ± 0.3 | 159.7 ± 19.0 | 148.1 ± 4.1 | 153.9 ± 15.6 | 525.6 ± 12.0 |
|  |  | Percentage | 11.4 ± 0.9 | 0.7 ± 0.1 | 30.4 ± 3.8 | 28.1 ± 1.0 | 29.3 ± 2.7 | 100 |
|  | 7 | Amount | 61.0 ± 6.9 | 3.7 ± 1.0 | 243.9 ± 6.1 | 175.1 ± 12.2 | 201.5 ± 11.6 | 685.2 ± 5.6 |
|  |  | Percentage | 8.9 ± 1.0 | 0.5 ± 0.1 | 35.6 ± 0.9 | 25.5 ± 1.7 | 29.4 ± 1.7 | 100 |
|  | 9 | Amount | 64.1 ± 5.2 | 4.0 ± 0.4 | 284.8 ± 24.9 | 265.7 ± 35.3 | 267.2 ± 14.9 | 886.0 ± 58.5 |
|  |  | Percentage | 7.3 ± 0.8 | 0.5 ± 0.1 | 32.1 ± 0.8 | 29.9 ± 2.0 | 30.3 ± 2.6 | 100 |
|  | 15 | Amount | 66.9 ± 4.8 | 4.9 ± 0.4 | 274.4 ± 16.1 | 231.6 ± 13.5 | 210.4 ± 17.1 | 788.3 ± 35.1 |
|  |  | Percentage | 8.5 ± 0.9 | 0.6 ± 0.1 | 34.8 ± 1.2 | 29.4 ± 1.5 | 26.7 ± 1.0 | 100 |
| Fanmai 5 | 1 | Amount | 50.6 ± 2.8 | 3.7 ± 0.6 | 64.3 ± 2.6 | 31.0 ± 4.4 | 231.8 ± 25.4 | 381.5 ± 28.1 |
|  |  | Percentage | 13.3 ± 0.8 | 1.0 ± 0.2 | 16.9 ± 1.7 | 8.1 ± 0.9 | 60.7 ± 2.4 | 100 |
|  | 3 | Amount | 67.9 ± 10.1 | 4.4 ± 0.6 | 87.5 ± 9.7 | 49.7 ± 4.8 | 420.9 ± 18.5 | 630.5 ± 26.7 |
|  |  | Percentage | 10.7 ± 1.2 | 0.7 ± 0.1 | 13.9 ± 1.1 | 7.9 ± 1.1 | 66.8 ± 2.2 | 100 |
|  | 5 | Amount | 66.5 ± 9.3 | 3.8 ± 1.8 | 99.0 ± 15.5 | 52.1 ± 8.3 | 509.4 ± 58.6 | 730.7 ± 35.7 |
|  |  | Percentage | 9.1 ± 1.3 | 0.5 ± 0.3 | 13.6 ± 2.8 | 7.2 ± 1.5 | 69.6 ± 4.5 | 100 |
|  | 7 | Amount | 70.8 ± 5.1 | 4.4 ± 0.4 | 121.8 ± 4.3 | 57.9 ± 5.0 | 574.8 ± 25.2 | 829.7 ± 28.4 |
|  |  | Percentage | 8.5 ± 0.8 | 0.5 ± 0.1 | 14.7 ± 0.6 | 7.0 ± 0.5 | 69.3 ± 0.8 | 100 |
|  | 9 | Amount | 81.9 ± 0.9 | 4.3 ± 0.2 | 289.8 ± 32.9 | 76.1 ± 3.2 | 682.9 ± 38.0 | 1135.0 ± 0.8 |
|  |  | Percentage | 7.2 ± 0.1 | 0.4 ± 0.1 | 25.5 ± 2.9 | 6.7 ± 0.3 | 60.1 ± 3.3 | 100 |
|  | 15 | Amount | 76.1 ± 7.3 | 4.6 ± 0.8 | 272.7± 14.7 | 73.6 ± 12.5 | 506.1 ± 33.3 | 933.1 ± 47.0 |
|  |  | Percentage | 8.2 ± 1.2 | 0.5 ± 0.1 | 29.2 ± 1.3 | 7.9 ± 1.0 | 54.2 ± 1.2 | 100 |
| *continued on next page* | | | | | | | | |
| *S2 Table continued from previous page* | | | | | | | | |
| Shanken 99 | 1 | Amount | 40.1 ± 7.0 | 2.5 ± 0.9 | 66.0 ± 11.7 | 43.7 ± 1.5 | 127.9 ± 11.5 | 280.2 ± 5.9 |
|  |  | Percentage | 14.2 ± 2.2 | 0.9 ± 0.3 | 23.6 ± 4.5 | 15.6 ± 0.9 | 45.6 ± 3.6 | 100 |
|  | 3 | Amount | 54.0 ± 5.4 | 3.2 ± 0.9 | 109.8 ± 11.9 | 46.3 ± 4.6 | 230.8 ± 22.7 | 444.0 ± 37.2 |
|  |  | Percentage | 12.2 ± 0.8 | 0.7 ± 0.2 | 24.7 ± 1.7 | 10.4 ± 0.9 | 52.0 ± 2.0 | 100 |
|  | 5 | Amount | 56.0 ± 6.7 | 3.7 ± 0.3 | 138.6 ± 19.2 | 53.3 ± 7.8 | 296.9 ± 11.6 | 548.5 ± 36.1 |
|  |  | Percentage | 10.2 ± 0.6 | 0.7 ± 0.1 | 25.2 ± 2.0 | 9.7 ± 1.0 | 54.2 ± 3.2 | 100 |
|  | 7 | Amount | 57.3 ± 8.5 | 4.1 ± 1.0 | 299.7 ± 28.6 | 95.2 ± 11.7 | 363.4 ± 21.7 | 819.7 ± 28.9 |
|  |  | Percentage | 7.0 ± 0.8 | 0.5 ± 0.1 | 36.5 ± 2.2 | 11.5 ± 1.2 | 44.4 ± 3.8 | 100 |
|  | 9 | Amount | 56.8 ± 10.5 | 4.4 ± 1.3 | 357.1 ± 30.0 | 99.6 ± 13.7 | 431.3 ± 19.0 | 949.2 ± 40.0 |
|  |  | Percentage | 5.9 ± 1.0 | 0.5 ± 0.1 | 37.6 ± 1.7 | 10.5 ± 1.0 | 45.5 ± 3.3 | 100 |
|  | 15 | Amount | 67.9 ± 10.3 | 4.0 ± 2.2 | 357.9 ± 14.3 | 99.9 ± 3.0 | 367.9 ± 19.4 | 897.7 ± 41.4 |
|  |  | Percentage | 7.5 ± 0.9 | 0.5 ± 0.3 | 39.9 ± 0.4 | 11.1 ± 0.4 | 41.0 ± 0.8 | 100 |

The wax amount was expressed in µg of wax per g of ear (dry weight). Mean values of total wax loads, amount and percentage (%) of individual compound classes are given with SD (*n* = 3).
