# Supplementary figures and images for: Correction: Developmental Changes in Composition and Morphology of Cuticular Waxes on Leaves and Spikes of Glossy and Glaucous Wheat (Triticum aestivum L.)
Source: PLoS One. 2015 Nov 18;10(11):e0143671. doi: 10.1371/journal.pone.0143671 (PMC4651351; doi:10.1371/journal.pone.0143671)

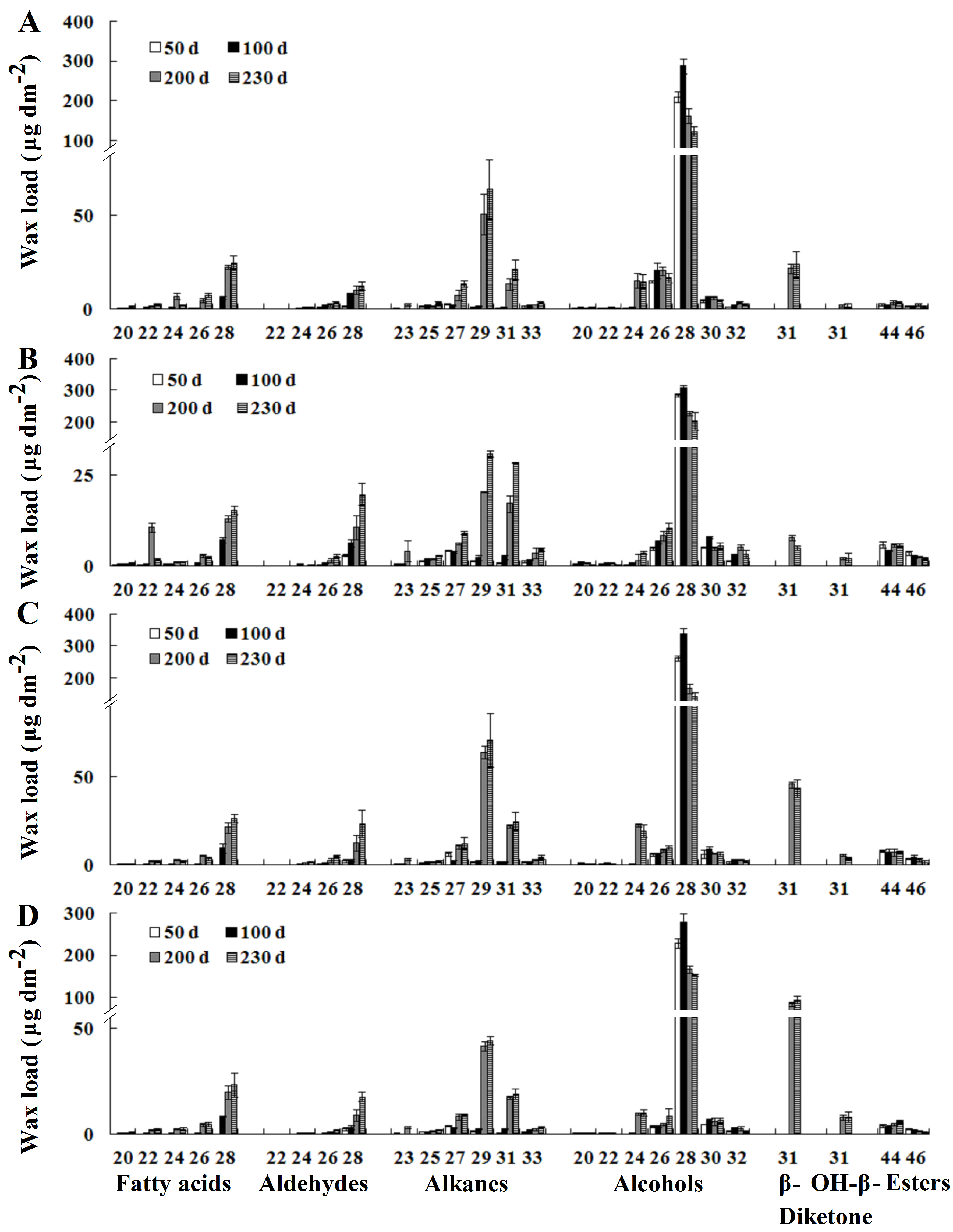

Supplement: S1 File — (ZIP) [file pone.0143671.s001.zip › Fig.3.TIF]

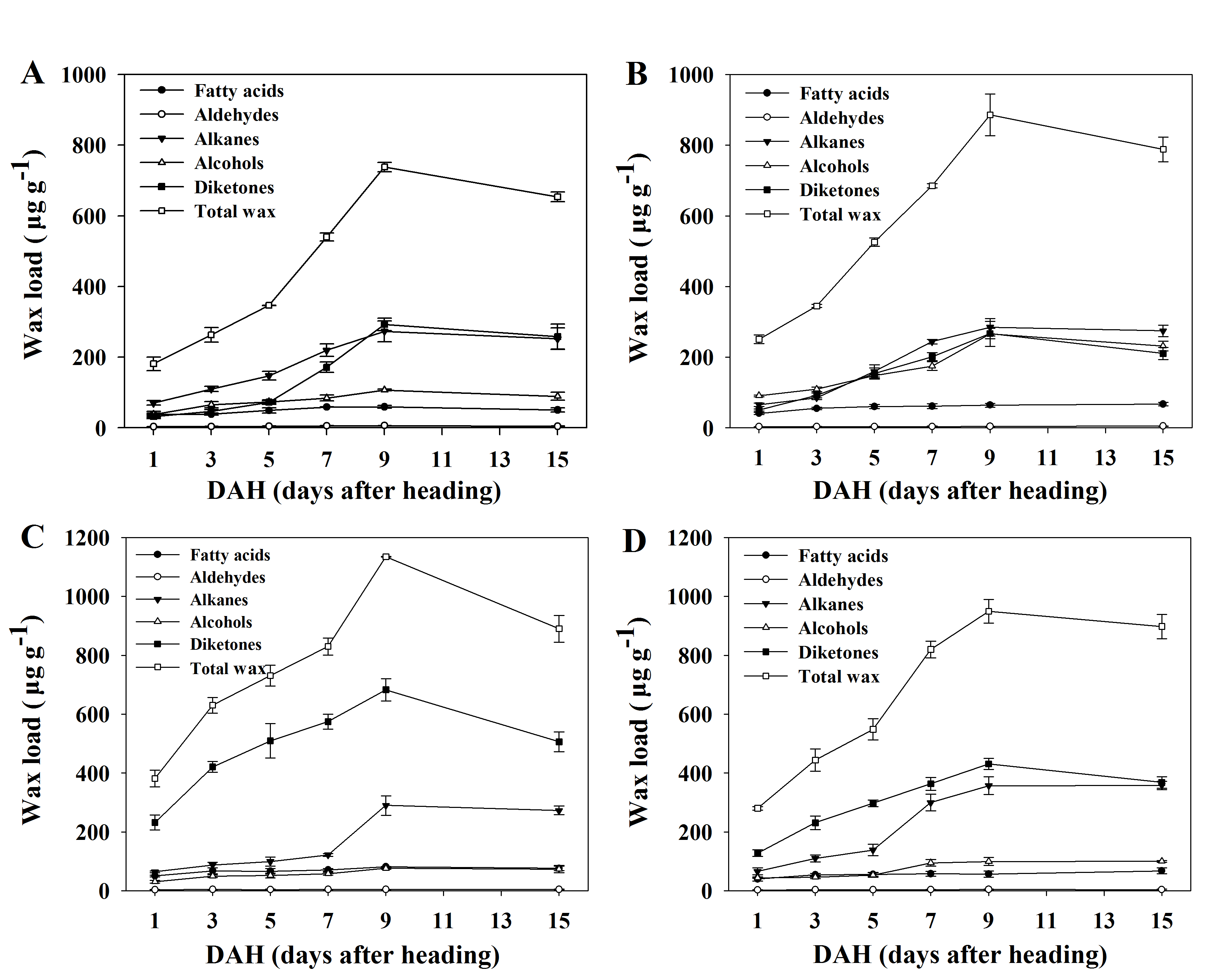

Supplement: S1 File — (ZIP) [file pone.0143671.s001.zip › Fig.4.TIF]

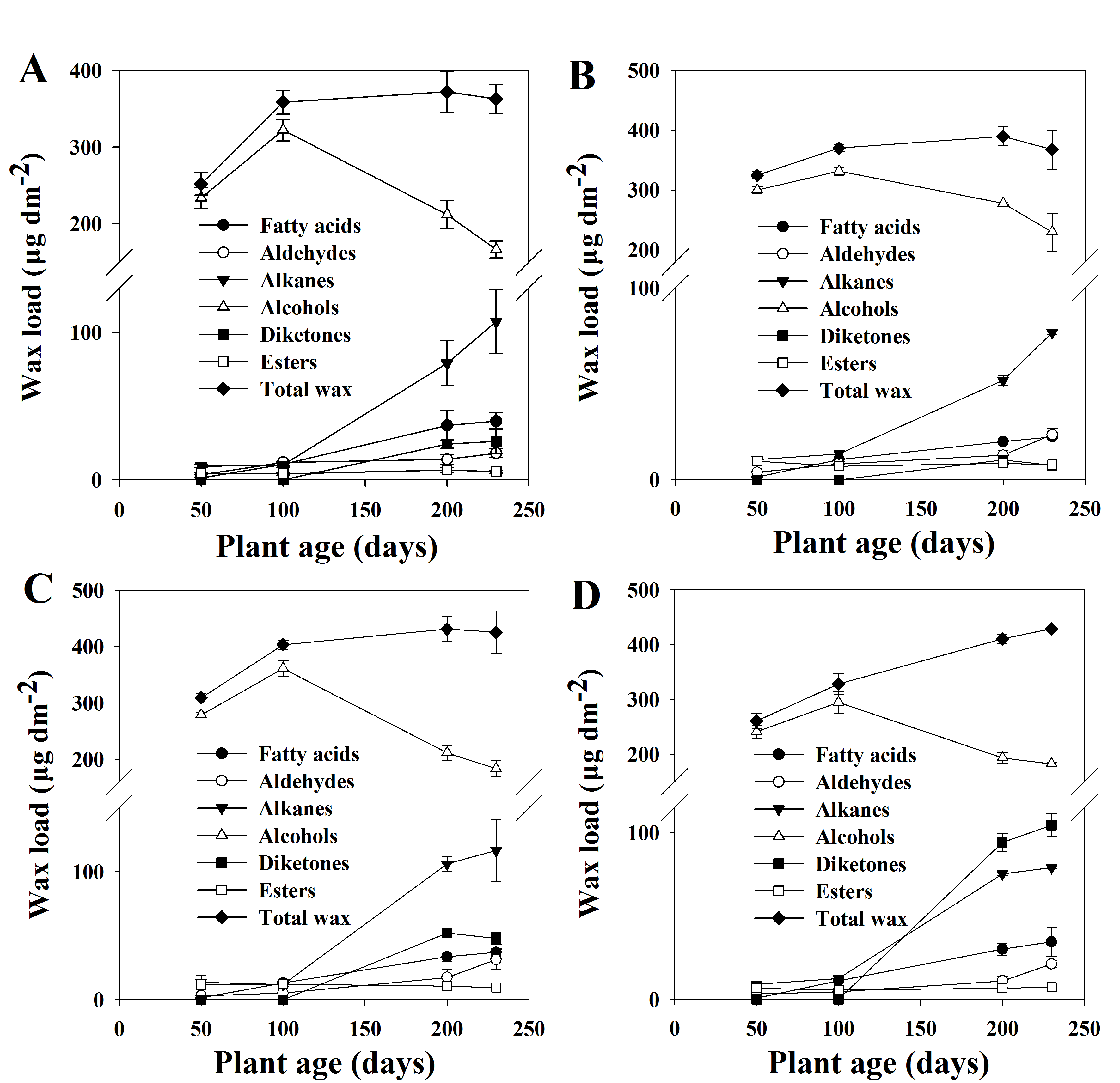

Supplement: S1 File — (ZIP) [file pone.0143671.s001.zip › Fig.2.TIF]

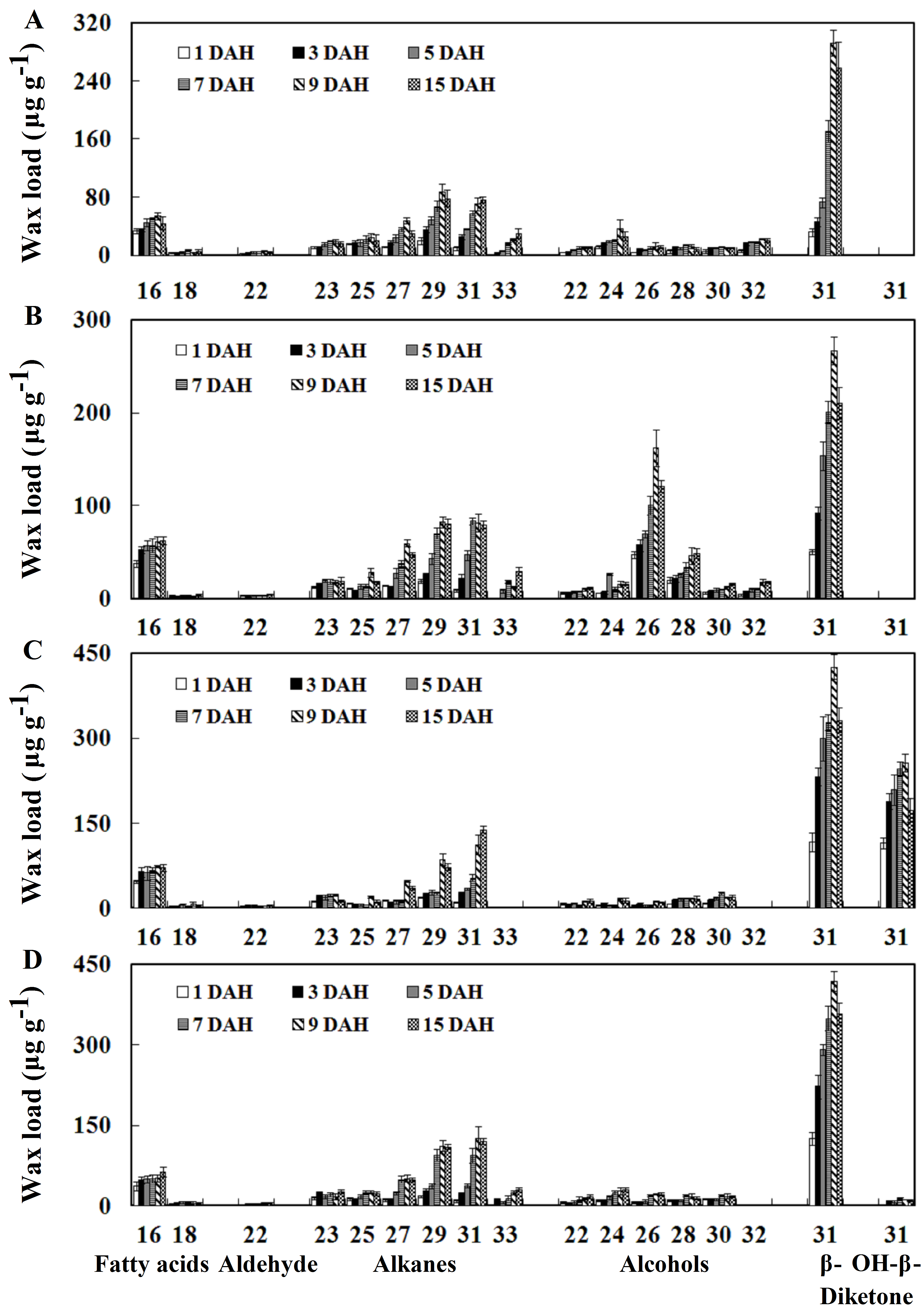

Supplement: S1 File — (ZIP) [file pone.0143671.s001.zip › Fig.5.TIF]

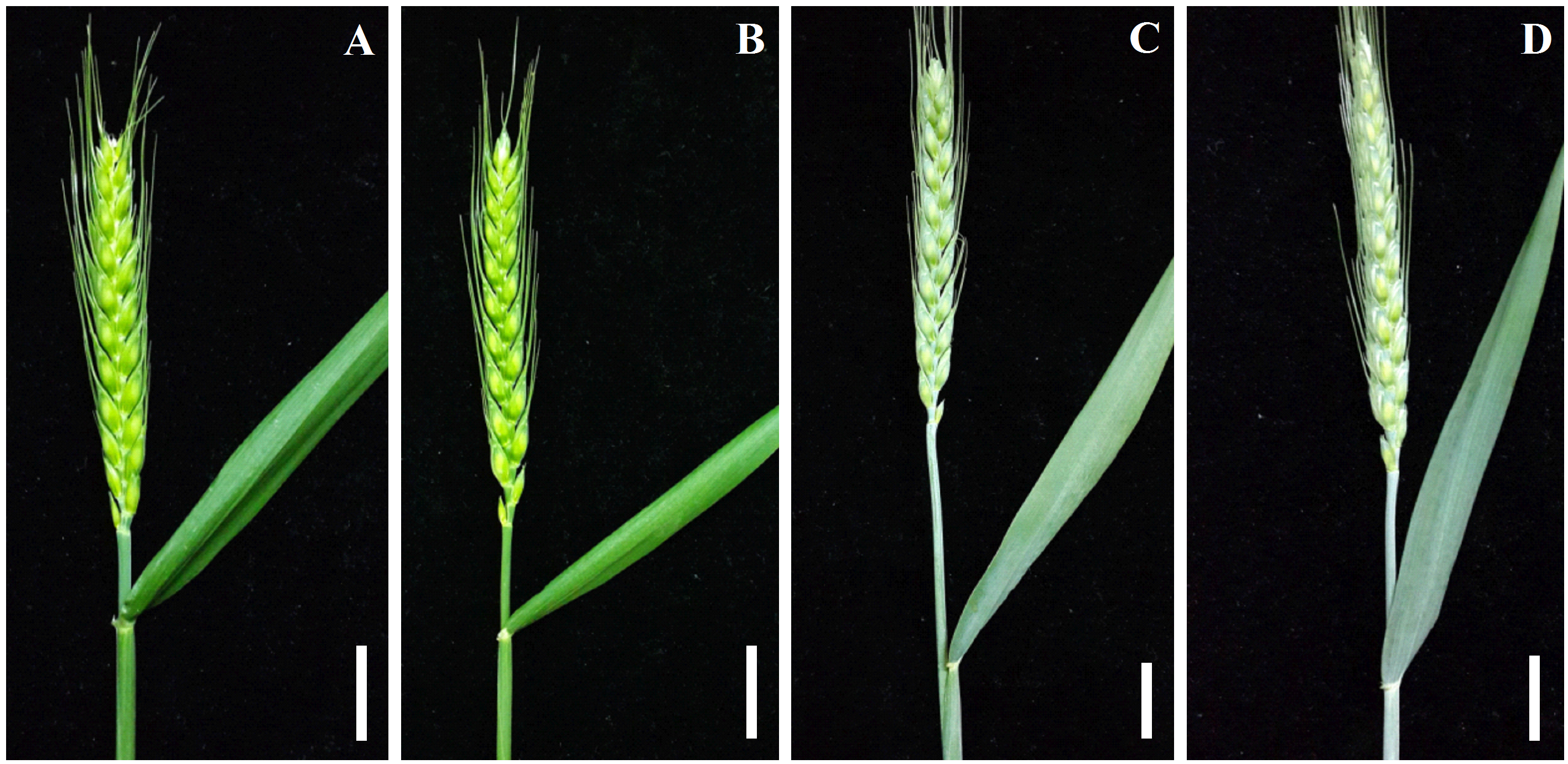

Supplement: S1 File — (ZIP) [file pone.0143671.s001.zip › Fig.1.TIF]

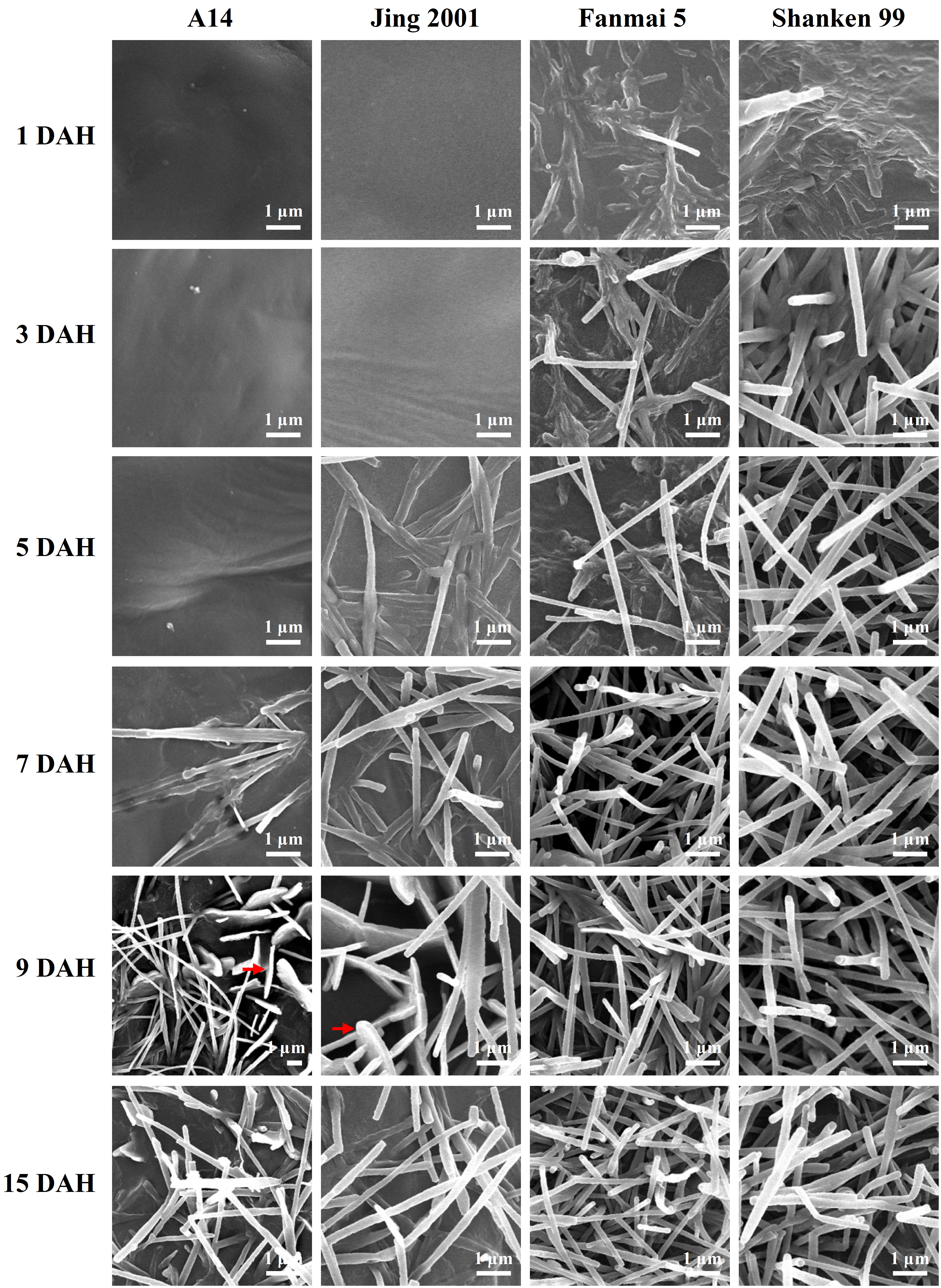

Supplement: S1 File — (ZIP) [file pone.0143671.s001.zip › Fig.6.TIF]
